# Advanced Near‐Infrared Light for Monitoring and Modulating the Spatiotemporal Dynamics of Cell Functions in Living Systems

**DOI:** 10.1002/advs.201903783

**Published:** 2020-02-27

**Authors:** Guangcun Chen, Yuheng Cao, Yanxing Tang, Xue Yang, Yongyang Liu, Dehua Huang, Yejun Zhang, Chunyan Li, Qiangbin Wang

**Affiliations:** ^1^ CAS Key Laboratory of Nano‐Bio Interface Division of Nanobiomedicine and *i*‐Lab CAS Center for Excellence in Brain Science Suzhou Institute of Nano‐Tech and Nano‐Bionics Chinese Academy of Sciences Suzhou 215123 China; ^2^ Suzhou Key Laboratory of Functional Molecular Imaging Technology Suzhou Institute of Nano‐Tech and Nano‐Bionics Chinese Academy of Sciences Suzhou 215123 China; ^3^ School of Nano‐Tech and Nano‐Bionics University of Science and Technology of China Hefei 230026 China; ^4^ College of Materials Sciences and Opto‐Electronic Technology University of Chinese Academy of Sciences Beijing 100049 China

**Keywords:** cell function sensing, living systems, molecular imaging, near‐infrared light, photoregulation, spatiotemporal dynamics

## Abstract

Light‐based technique, including optical imaging and photoregulation, has become one of the most important tools for both fundamental research and clinical practice, such as cell signal sensing, cancer diagnosis, tissue engineering, drug delivery, visual regulation, neuromodulation, and disease treatment. In particular, low energy near‐infrared (NIR, 700–1700 nm) light possesses lower phototoxicity and higher tissue penetration depth in living systems as compared with ultraviolet/visible light, making it a promising tool for in vivo applications. Currently, the NIR light‐based imaging and photoregulation strategies have offered a possibility to real‐time sense and/or modulate specific cellular events in deep tissues with subcellular accuracy. Herein, the recent progress with respect to NIR light for monitoring and modulating the spatiotemporal dynamics of cell functions in living systems are summarized. In particular, the applications of NIR light‐based techniques in cancer theranostics, regenerative medicine, and neuroscience research are systematically introduced and discussed. In addition, the challenges and prospects for NIR light‐based cell sensing and regulating techniques are comprehensively discussed.

## Graphical abstract

NIR light‐based technique, including optical imaging and photoreglation, has become one of the most important tools for both fundamental research and clinical practice. Herein, the recent progress of NIR light‐based cell function sensing and modulation in cancer theranostics, regenerative medicine, and neuroscience research is summarized.

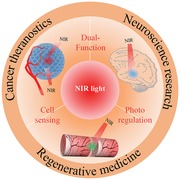

## 1 Near‐Infrared Light and Its Biomedical Applications

Light is an essential physical element that governs a wide range of essential physiological processes in almost all living organisms, including sensing, energy capturing, and metabolism. For instance, animals capture light signals through their eyes to sense the environment. Moreover, plants and microorganisms obtain energy from sunlight through photosynthesis. Due to the critical role of light in our lives, it has inspired scientists to explore the interaction between light and organisms. Consequently, the increased understanding of light‐sensing and light‐based energy capture in organisms also have stimulated the development of light‐based technologies for numerous biomedical applications, including biosensing and light‐based regulation.^[^ 1, 2, 3, 4, 5 ^]^ As compared with other imaging and regulation modalities (magnetic resonance, ultrasound, etc.), light can serve as a noninvasive tool to monitor and regulate the spatiotemporal dynamics of cell signaling in living systems with a micron spatial resolution and a sub‐millisecond temporal resolution.^[^ 6, 7, 8, 9 ^]^ Currently, light has been extensively used in both fundamental research and clinical practice, such as cell signal sensing, enzyme activity monitoring, controlled drug release, visual regulation, neuromodulation, cancer diagnosis and treatment.^[^ 1, 5, 6, 8, 10 ^]^

**Figure 1:**
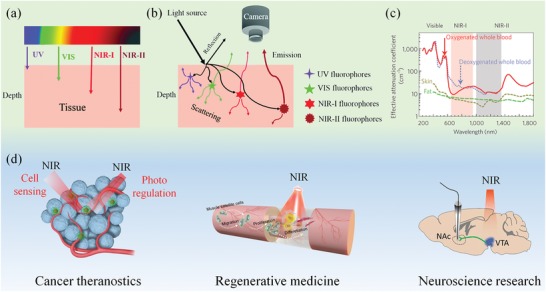
Light–tissue interactions and biomedical applications of NIR light. a) The penetration depth of ultraviolet (UV), visible (vis), and NIR light in tissues. As compared with UV/vis and NIR‐I lights, NIR‐II light exhibits the deepest penetration depth in tissues. b) Schematic illustration of in vivo imaging using fluorophores with different wavelengths. In addition to the tissue penetration depth, NIR‐II light also exhibits the least scattering compared with UV/vis and NIR‐I fluorophores. Reproduced with permission.^[^ 12 ^]^ Copyright 2018, The Royal Society of Chemistry. c) Effective attenuation coefficient of various biological entities (oxygenated and deoxygenated whole blood, skin, and fat) over optical wavelengths from 200 to 1800 nm. The absorption and scattering of the biological components in the NIR window (including NIR‐I and NIR‐II) are significantly lower than that in the UV/vis window. Reproduced with permission.^[^ 11 ^]^ Copyright 2009, Nature Publishing Group. d) NIR light‐based cell function sensing and regulating in cancer theranostics, regenerative medicine, and neuroscience research. Left: Reproduced with permission.^[^ 26 ^]^ Copyright 2019, Wiley‐VCH. Middle: Reproduced with permission.^[^ 27 ^]^ Copyright 2019, American Chemical Society. Right: Reproduced with permission.^[^ 28 ^]^ Copyright 2018, American Association for the Advancement of Science.

Light can interact with biological tissues in many ways. Once entering the tissue, light will generally be reflected, absorbed or scattered by molecules within the tissue (**Figure** **1**a–c). It has been found that the wavelength of the light is a key issue involved in the interaction of light and organisms.^[^ 8, 11, 12 ^]^ Generally, light with a longer wavelength possesses lower energy, thus the reaction activity of long‐wavelength light with most biological tissues is much lower than that of short‐wavelength light. The visible light with a wavelength of 400–700 nm has been extensively used in biomedical researches.^[^ 3, 4, 13 ^]^ Due to the strong interaction between visible light and tissues, visible light faces several critical challenges, such as its shallow tissue penetration depth (<1 mm), the strong autofluorescence of mammalian tissues, and high phototoxicity.^[^ 8, 14, 15 ^]^ These shortcomings greatly limit the applications of visible light in deep tissues of living organisms. In comparison with visible light, near‐infrared (NIR, 700–1700 nm) light has longer wavelength and lower energy, which possesses a much less tissue scattering and absorption in biological tissues (Figure 1a–c).^[^ 8, 12, 16, 17, 18, 19 ^]^ Thereby, the NIR window has been found as a biological transparency window with minimal photodamage and deep tissue penetration depth (Figure 1a). In more recent studies, a penetration depth of more than one centimeter, a spatial resolution of tens of microns and a temporal resolution of milliseconds have been achieved by using the NIR light in the second NIR window (NIR‐II, 1000–1700 nm) because of the minimal absorption and scattering of NIR‐II light in biological tissues.^[^ 20, 21, 22, 23, 24, 25 ^]^ Given these unique advantages of NIR light, numerous NIR fluorophores and NIR‐responsive molecules have been developed for bioimaging and manipulating cell functions with exceptional spatiotemporal precision and sensitivity in deep tissues.^[^ 5, 6, 8, 12 ^]^

For bioimaging and biosensing, fluorescent agents can mainly be divided into endogenous genetical encoded reporter and exogenous fluorescent agents. Nowadays, the fluorescent protein‐based fluorescence imaging (FI) and luciferase‐based bioluminescence imaging (BLI) have been the most important tools for cell imaging and sensing.^[^ 3, 29, 30, 31 ^]^ These fluorescence reporters can be encoded by their corresponding genes and be passed in generations, thus offering a stable fluorescence reporter in living cells and animals. Furthermore, the expression of these fluorescence reporters can also be controlled by specific promoter and thus in vivo dynamic reporting cell signals.^[^ 32, 33 ^]^ Recently, a series of NIR‐emitting fluorescent proteins with an emission region of 650–800 nm have been developed and successfully applied in bioimaging with high signal‐to‐background ratio and sensitivity.^[^ 34, 35, 36, 37 ^]^ For example, the first NIR‐emitting fluorescent protein was developed by Shu and colleagues using a bacterial phytochrome scaffold (DrBphP). The bright fluorescent protein, IFP1.4, can emit in the NIR‐I window with a peak emission of 708 nm.^[^ 35 ^]^ In another example, Iwano and colleagues established a highly sensitive and stable AkaLumine/Akaluc‐based bioluminescence (AkaBLI) with an engineered firefly luciferase and a red‐shifted luciferin analog. The AkaBLI possesses an emission peak of 677 nm and can be used to monitor single cell in deep tissues of freely moving animals.^[^ 38 ^]^ Unlike endogenous genetical encoded reporters, the wavelength of the exogenous NIR probe can be extended to any NIR range from 700–1700 nm.^[^ 8, 22 ^]^ Currently, numerous types of fluorescent agents have been developed, including small molecular dyes, organic nanoprobes and inorganic nanoprobes.^[^ 8, 12, 17 ^]^ With a rational design, these NIR agents can respond to specific physiological microenvironments in cells (such as pH, temperature, redox status, and membrane potential), thus reporting the physiological status of cells, including apoptosis, viability, cell signaling, and neural activity.^[^ 17, 39, 40 ^]^ In addition, fluorescent agents are able to be functionalized with biomolecules which can target and respond to specific proteins, nucleic acid, lipid or other cell metabolites, thus reporting the cell signaling, enzyme activity, and other cell metabolism processes in cells.^[^ 40, 41 ^]^

In addition to cell sensing, NIR light also has generated great interest among researchers for their feasibilities in cell control and modulation with exceptional spatiotemporal precision and sensitivity.^[^ 6, 42, 43 ^]^ The NIR light can penetrate into the deep tissues (>1 cm) and can be directed to specific subcellular locations with micron spatial resolution, and can be turned on/off with a sub‐millisecond temporal resolution, thereby offering a noninvasive wireless tool to control specific cells in vivo.^[^ 5, 6 ^]^ Currently, optogenetic technologies using light‐sensitive ion channels, such as Channelrhodopsin (ChR),^[^ 44 ^]^ Archaerhodopsin (Arch),^[^ 45 ^]^ and Halorhodopsin (NpHR),^[^ 46 ^]^ are the most powerful and popular tools for cell modulation. The development of NIR light‐sensitive proteins, such as the red‐shifted variant of channelrhodopsin (ReaChR)^[^ 47 ^]^ and bacterial phytochromes (BphPs),^[^ 34 ^]^ has made it possible to perform optogenetic research using red or NIR light. More recently, the application of NIR light activatable nanomaterials have greatly extended the application of NIR light for photoregulation in biomedicine via several mechanisms. First, upconversion nanoparticles (UCNPs) can be excited by NIR light, for example, 980 nm or longer wavelength, and emit ultraviolet (UV) or visible light that can active light‐sensitive protein‐ion channels in genetically engineered cells, offering a possibility of operating optogenetic researches with high tissue penetration depth NIR‐II light.^[^ 28, 48 ^]^ Second, a series of photothermal nanomaterials, including Au nanomaterials and organic semiconducting nanomaterials, are capable of generating local heat in cells during NIR light irradiation.^[^ 49, 50 ^]^ Thereby, a series of temperature‐dependent biological processes can be modulated by the generated heat, such as photothermal cell killing, heat‐related gene expression, heat‐controlled drug release, and photothermal neuron activation.^[^ 27, 49, 51, 52 ^]^ Third, NIR light can be used to generate reactive oxygen species (ROS) via the photosensitizer‐based nanomaterials, thus being widely used in photodynamic therapy (PDT) of cancer.^[^ 26, 53 ^]^ Fourth, NIR light also can be used to modulate electron transfer in cells via the photoelectrochemical nanomaterials (such as silicon‐based nanostructured materials), thus being used for optical neuromodulation and stem cell‐based regenerative medicine.^[^ 54, 55 ^]^

In this review, we summarize the recent progress of NIR light‐based cell function sensing and modulating in cancer theranostics, regenerative medicine, and neuroscience research (Figure 1d). In particular, the recent breakthrough researches of cell imaging and cell regulation in the NIR‐II region are emphasized and discussed. In addition, the challenges and prospects for NIR light‐based cell sensing and regulating techniques are comprehensively discussed.

## 2 Advanced NIR Light for Cell Function Imaging

Due to the minimal scattering and absorption of NIR light by tissues, and the minimal autofluorescence of tissues in the NIR region, an in vivo deep‐tissue imaging with high spatiotemporal resolution can be achieved by using NIR imaging.^[^ 11, 16, 19 ^]^ Given these advantages, NIR imaging has been extensively applied in sensing the apoptosis, viability, differentiation, membrane potential, gene expression, enzyme activity, and cell metabolism of numerous types of cells.^[^ 40, 56 ^]^ These studies have greatly promoted the development of numerous fields, such as cancer theranostics, stem cell‐based regenerative medicine and neuroscience (**Table** **1**).

**Table 1:** Representative researches of cell function sensing using NIR probes

Research field	Probes	Excitation/emission [nm/nm]	Mechanisms	Applications	Reference
Cancer diagnosis	KcapQ	647/500–750 (peak 665)	A fluorophore‐quencher pair (Alexa Fluor 647 and QSY 21) linked by DEVD peptide	Sensing apoptosis in HeLa cells	^[^ 57 ^]^
	1‐RGD	780/810 & photoacoustic signal	Turn on the fluorescence of ICG via cleaving the DEVD peptide by caspase	Reporting of caspase‐3 activity and distribution within tumor tissues in mice	^[^ 58 ^]^
	Pt^IV^ probe UCNPs@SiO_2_	620/640–800 (peak 665)	Turn on the fluorescence of Cy5 via cleaving the DEVD peptide by caspase	Photoactivation of antitumor prodrug and simultaneous cellular apoptosis imaging	^[^ 59 ^]^
	AuHNRs‐DTPP	488/500–700	Release of DTPP from AuHNRs‐DTPP via caspase treatment	NIR‐II PDT, PTT and real‐time apoptosis imaging of tumor	^[^ 50 ^]^
	Cy‐annexin V	635/650–800	Cy5.5 conjugated Annexin V target to apoptotic cells	Imaging apoptosis of gliosarcoma	^[^ 60 ^]^
	Symmetrical squaraine (Sq)‐based fluorescent probe	670/690; 400/560	Michael addition reaction between Sq and thiol of GSH	Sensing GSH in apoptotic HepG2 and 3T3L1 cells	^[^ 61 ^]^
	HISSNPs	808/900–1500 (peak 1070)	Fluorescence turns on by hyaluronidase and GSH	In vivo imaging MCF‐7 cancer xenografts in mice	^[^ 62 ^]^
	UC‐PH1/H2	514/545–580; 640–700	Hybridization chain reaction	Reporting the c‐MYC mRNA in L‐02 cells, HeLa cells, and MCF‐7 cells	^[^ 63 ^]^
	PbS/CdS/ZnS core/shell/shell QDs	910/1200	Fluorescence turned on by matrix metalloproteinase (MMP)	In vivo imaging the colon cancer in mice	^[^ 64 ^]^
	PCyFF‐Gd	710/680	Fluorescence turned on by alkaline phosphatase (ALP)	Imaging of HeLa tumors and orthotopic liver tumors in mice	^[^ 65 ^]^
	Nano‐PT	785/900–1300	Hydrogen sulfide (H_2_S)‐activatable probe through the thiol‐halogen nucleophilic substitution between BODIPY and H_2_S	H_2_S‐activated NIR‐II fluorescence to guide PTT of colorectal cancer	^[^ 66 ^]^
	IR1048‐MZ	980/1046	Hypoxia‐triggered NIR‐II fluorescence	In vivo imaging and PTT of A549 tumor in mice	^[^ 67 ^]^
	V&A@Ag_2_S	808/1200	ONOO^−^‐activatable NIR‐II probes	Real‐time assessment of the pathological process of TBI	^[^ 68 ^]^
Regenerative medicine	DL2	665/700; 480/530	Release Cy5.5 or FITC by intracellular proteases or ROS	Imaging the apoptosis of hMSCs transplanted in the dermis of the mouse ear pinna	^[^ 69 ^]^
	Ag_2_S QDs & RFLuc	808/1200; 620 bioluminescence	Dual‐modal BLI/NIRFI imaging	The fate of transplanted stem cells in a mouse model of acute liver failure	^[^ 70 ^]^
	miRFP670, miRFP703 and miRFP709	635/670, 703, 709	IkBa reporter‐controlled red fluorescence protein	NF‐κB pathway in cultured cells	^[^ 36 ^]^
	PLL‐coated MB nanosensor	485/515; 550/570	Sensing ALP and GAPDH mRNA via complementary target oligonucleotides	Monitor the osteogenic differentiation of mesenchymal stem cells	^[^ 71 ^]^
	Multifunctional nanocomplex	490/500–700 (peak 530); 550/550–700 (peak 570)	Release of Alexa488 tag or Cy3 tag from Au NPs after reaction with the target Tubb3 or Fox3 mRNA	Monitor the Tubb3 or Fox3 mRNA activities in NSCs at specific differentiation stages	^[^ 72 ^]^
	Ag_2_S QDs & RFLuc, GLuc	808/1200; 620 and 480 bioluminescence	NIR‐II fluorescence/dual BLI multiplexed imaging	Report the distribution, viability, and osteogenic differentiation of transplanted stem cells in a calvarial defect mouse model.	^[^ 32 ^]^
Neural activity monitoring	QuasAr3	640/660–740 (peak 720)	Voltage‐dependent illumination of QuasAr3 due to the protonation of the Schiff base in a 13‐cis photocycle intermediate	Real‐time record the supra‐ and subthreshold voltage dynamics in multiple neurons of behaving mice	^[^ 73 ^]^
	ICG	780/818–873	Electrochromic nature of ICG and the redistribution of ICG within or around the membrane	Sensing the action potential of neurons; monitor cardiac electrical activity	^[^ 74, 75 ^]^
	FlareFRET	A series of dyes with emission from 500 to 700 nm	Electrochromic fluorescence resonance energy transfer (eFRET) between rhodopsin and organic dyes	Optical mapping of electrical connections between cultured cells	^[^ 76 ^]^
	QD‐peptide‐fullerene bioconjugation	405/605	Voltage‐sensitive electron transfer (ET) between QDs and fullerene	Cellular membrane potential imaging of neurons	^[^ 77 ^]^

### 2.1 NIR Light‐Based Molecular Imaging in Cancer Research

Fluorescence imaging has become one of the most important techniques for cancer research.^[^ 16, 17 ^]^ In fundamental cancer researches, the understanding of the processes and underlying mechanisms of cancer development by fluorescence imaging is vital for developing efficient cancer treatment. In addition, fluorescence imaging has also been widely used for imaging‐guided surgery of tumor in clinical practices.^[^ 78 ^]^ Nowadays, a series of fluorescence molecular imaging techniques have been developed for sensing the apoptosis, cell signaling and microenvironment characteristics of tumor.^[^ 16, 17, 40 ^]^

#### 2.1.1 Apoptosis and Necrosis Imaging of Cancer Cells

Cell apoptosis is a programmed cell death that controls multiple biological processes in the living organisms, such as tissue self‐renewal, immunity, disease occurrence.^[^ 79 ^]^ Thus, cell apoptosis imaging has attracted great attentions among researchers due to its great potential for disease diagnosis and imaging‐guided disease treatment.^[^ 60, 80 ^]^ For example, enhanced cell apoptosis is a characteristic of acquired immunodeficiency syndrome, liver diseases, and Alzheimer's and Parkinson's disease.^[^ 81, 82 ^]^ Thus, cell apoptosis imaging can serve as an effective tool for disease diagnosis. On the other hand, cell apoptosis imaging can report the status of numerous cells such as cancer cells, thus can be used to evaluate the therapeutic efficacy of cancer therapy.

**Figure 2:**
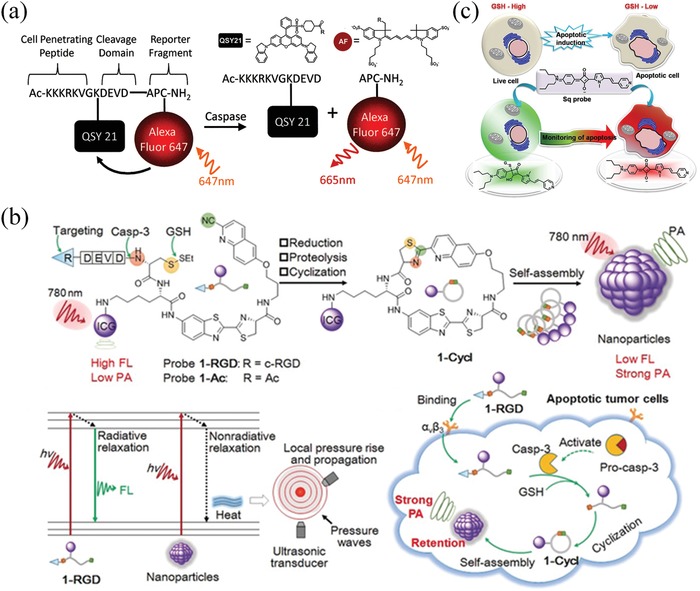
NIR probes for cell apoptosis sensing. a) A cell‐penetrating and caspase‐activatable NIR probe constructed using a fluorophore (Alexa Fluor 647)‐quencher (QSY 21) pair. Reproduced with permission.^[^ 57 ^]^ Copyright 2009, American Chemical Society. b) A photoacoustic and NIR probe for tumor apoptosis imaging. Chemical structures of 1‐RGD and 1‐Ac and their chemical conversion during apoptosis sensing. Reproduced with permission.^[^ 58 ^]^ Copyright 2019, Wiley‐VCH. c) The Sq‐based fluorescent probe for real‐time sensing intracellular GSH in apoptosis tumor cells. Reproduced with permission.^[^ 61 ^]^ Copyright 2017, Wiley‐VCH.

Nowadays, a series of cell apoptosis imaging strategies have been developed to monitor certain apoptotic phenotypes such as caspase activation,^[^ 57 ^]^ phosphatidylserine externalization,^[^ 60 ^]^ DNA fragmentation^[^ 83 ^]^ and variated physiological microenvironments (redox status, pH, etc.).^[^ 61 ^]^ The apoptosis‐associated caspase activity is the most commonly used indicator for apoptosis detection.^[^ 50, 57, 59 ^]^ Generally, a caspase‐sensing probe can be developed by linking a fluorophore and a quencher with a cleavable caspase‐3 substrate peptide, DEVD (Asp‐Glu‐Val‐Asp), to form a fluorophore‐quencher pair. In these caspase‐activatable NIR probes, once the DEVD is cleaved, fluorophore will be released and the fluorescence will be turned on to report the activity of caspase. For example, Maxwell et al. synthesized a cell‐penetrating NIR probe by sequentially linking a cell‐penetrating Tat peptide, a quencher‐QSY 21, a DEVD peptide and a fluorophore‐Alexa Fluor 647 (**Figure** **2**a). The caspase‐activatable NIR probe was successfully applied in sensing apoptosis in HeLa cells treated with doxorubicin.^[^ 57 ^]^ In 2019, Wang et al. reported a caspase‐responsive photoacoustic (PA) probe (1‐RGD) for real‐time imaging of tumor apoptosis in vivo.^[^ 58 ^]^ The 1‐RGD consists of a NIR dye (indocyanine green, ICG), a glutathione (GSH)‐reducible disulfide bond, a DEVD peptide substrate, a 2‐cyano‐6‐hydroxyquinoline (CHQ) and a D‐cysteine (D‐Cys) residue. The thiol and amino groups of the D‐Cys residue in 1‐RGD could be uncaged after reaction with intracellular GSH and active caspase‐3. After that, the free D‐Cys and CHQ subsequently formed a rigid and hydrophobic product 1‐cycl and consequently self‐assembled into nanoparticles. Thereby, the NIR fluorescence of ICG in nanoparticles was quenched via the aggregation‐caused quenching effect while the PA signal of ICG was distinctly enhanced via augmenting nonradiative relaxation processes (Figure 2b).

Phosphatidylserine externalization is another typical characteristic of apoptosis.^[^ 60 ^]^ Thus, the phosphatidylserine‐targeting molecule, Annexin V, has been widely used for developing cell apoptosis sensing probes. For example, Petrovsky et al. synthesized a Cy5.5 conjugated Annexin V (Cy‐annexin V) probe that can be used to image cell apoptosis in gliosarcoma by using near infrared fluorescence (NIRF) imaging.^[^ 60 ^]^ In this study, an active Cy‐annexin probe that bound to apoptotic Jurkat T cells was prepared and two to three times increase of NIRF signal in the treated tumor was observed with the active Cy‐annexin V probe. In addition, the NIRF signal in tumor could be observed by 75 min after active Cy‐annexin injection and remained for more than 20 h.

The disordered intracellular redox status is also considered to be a typical feature of cell apoptosis. Because GSH is the primary contributor to intracellular redox state, the depletion of GSH that generally occurred in the beginning of the cell death can promote the generation of ROS and activate the cell death machinery.^[^ 61, 84 ^]^ Thereby, intracellular glutathione (GSH) has also been used as a marker molecule that involved in cell apoptosis. In 2017, Saranya et al. reported a symmetrical squaraine (Sq)‐based fluorescent probe for real‐time sensing intracellular GSH that reduced in apoptosis tumor (Figure 2c). Because the cyclobutene ring of the Sq moiety can reactive with thiol via Michael addition reaction and the absorption and emission spectra of Sq is distinctly changed after the chemical activation. Thus, the NIR squaraine dye is capable of sensing GSH in a ratiometric manner. In this study, it was found that the absorption of Sq was switched from 670 to 400 nm and the emission of Sq was switched from 690 to 560 nm after interaction with GSH, so that real‐time report of apoptotic process in HepG2 and 3T3L1 cells was achieved.^[^ 61 ^]^ More recently, hyaluronidase and thiols cooperatively activatable NIR‐II probe was developed by Fan's group.^[^ 62 ^]^ The developed NIR‐II probe possesses an emission peak of 1064 nm and its fluorescence could be turned on by hyaluronidase and GSH, therefore to report the location of MCF‐7 cancer xenografts in vivo. With the excellent NIR fluorescence, the probe is also potential for sensing cell apoptosis in deep tissues.

#### 2.1.2 Sensing Other Cell Physiologies in Cancer Microenvironment

The unique characteristics of tumor cells and tumor microenvironment provide the possibility for specific detection of tumors. Nowadays, numerous markers of cancer have been identified, such as specific gene expression, enzyme activity, pH, redox, and hypoxia.^[^ 40, 85 ^]^

For gene expression detection in cancer cell, Li and coworkers developed a NIR light‐initiated hybridization chain reaction (HCR) approach for messenger RNA (mRNA) detection in cancer cells.^[^ 63 ^]^ In this study, one of the hairpins in the functional unit of the HCR was engineered by adding a short base tail at the 5′‐end via a photocleavable linker. By using the upconversion photochemistry, the HCR was initiated by NIR light irradiation, thus reporting the mRNAs in specific organelles of cells. In an example of cancer biomarker gene detection, the NIR light‐activated HCR was successfully used for reporting the c‐MYC mRNA in L‐02 cells, HeLa cells, and MCF‐7 cells.

**Figure 3:**
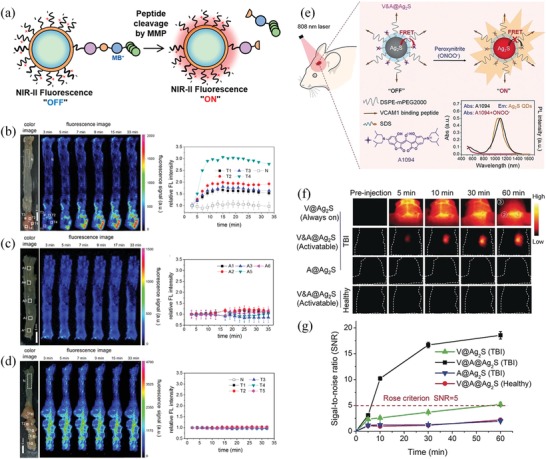
Disease microenvironment‐sensitive NIR‐II QDs for in vivo imaging. a) PbS/CdS/ZnS core/shell/shell QDs for sensing the activity of MMP in tumor microenvironment. b) Ex vivo fluorescence images of colon cancer by spraying with the activatable NIR QDs. c) Ex vivo fluorescence images of a normal healthy colon by spraying with the activatable NIR QDs. d) Ex vivo fluorescence images of colon cancer by spraying with the control NIR QDs. Reproduced with permission.^[^ 64 ^]^ Copyright 2017, American Chemical Society. e) Scheme of the V&A@Ag_2_S probe for detecting ONOO^−^ in vivo. f) NIR‐II fluorescence images of brain in TBI mice and healthy mice. g) Signal‐to‐noise ratios determined by the NIR‐II fluorescence imaging in (f). Reproduced with permission.^[^ 68 ^]^ Copyright 2019, Wiley‐VCH.

The high activity of matrix metalloproteinase (MMP) is known as a hallmark of the cancer microenvironment.^[^ 64, 86, 87 ^]^ Recently, Kim and colleagues reported an MMP‐activatable NIR‐II quantum dots (QDs) for tumor detection (**Figure** **3**a–d).^[^ 64 ^]^ In this study, the authors synthesized PbS/CdS/ZnS core/shell/shell QDs with NIR‐II emission. Then, methylene blue was conjugated to the QDs via a protease‐cleavable peptide sequence. Thus, the fluorescence of QDs could be quenched by the methylene blue via a photoexcited electron transfer (PET) quenching process. Once the linked peptide was cleaved by MMP, methylene blue (MB) was released and the fluorescence of QDs was turned on. In a colon cancer mouse model, the activatable QD probe showed selective fluorescence activation at tumor sites with high resolution. The MMP‐activatable NIR‐II QDs are particularly useful for in vivo detection of tumors in the deep tissue due to the high tissue penetrations of the NIR‐II imaging. In addition to MMP‐activatable probes, the alkaline phosphatase (ALP)‐activatable NIR probes also have been developed by Ye, et al.^[^ 65 ^]^ In this study, the synthesized probe (named PCyFF‐Gd) was activated by endogenous ALP and assembled on cell membranes, thereby turning on NIR fluorescence and magnetic resonance (MR) signals. Thus, the ALP‐activatable probe could accurately report the location of HeLa tumors and orthotopic liver tumors in vivo with high sensitivity.

In addition to the enzyme activity, tumor microenvironment‐responded NIR probes have also been reported for tumor detection, such as hydrogen sulfide (H_2_S)‐activatable probes, hypoxia‐triggered probes and peroxynitrite (ONOO^−^)‐activatable probes.^[^ 40, 66, 67, 68 ^]^ For example, the ONOO^−^ is a type of reactive nitrogen species (RNS) that abnormally generated and accumulated in the occurrence of multiple diseases, including cancer and inflammation.^[^ 68, 88, 89, 90 ^]^ Recently, the ONOO^−^‐activatable NIR‐II probes have been developed for reporting multiple diseases in vivo.^[^ 88 ^]^ For example, Wang and co‐workers synthesized a ONOO^−^‐activatable NIR‐II QD for real‐time monitoring the pathological process of traumatic brain injury (TBI) (Figure 3e–g).^[^ 68 ^]^ In this system, the fluorescence of Ag_2_S QDs were quenched in the intact V&A@Ag_2_S due to the fluorescence resonance energy transfer (FRET) between Ag_2_S QDs and the A1094 chromophore. After reaction with ONOO^−^, the NIR‐II fluorescence of Ag_2_S QDs was rapidly recovered due to the bleaching of A1094 by ONOO^−^. In a TBI model, this ONOO^−^‐activatable nanoprobe provided a convenient approach for in vivo real‐time assessment of the pathological process of TBI with high specificity and high sensitivity. We expected that this strategy is highly promising for other ONOO^−^‐related diseases detection.

### 2.2 NIR Imaging for Cell Function Sensing in Regenerative Medicine

Stem cell‐based regenerative medicine has shown its great potential for treating numerous diseases that are traditionally incurable.^[^ 91, 92 ^]^ The behaviors of transplanted stem cells, including migration, survival, paracrine effect, and differentiation, are vital for the safety and therapeutic efficacy of stem cell‐based therapy. Therefore, tracking and understanding the fate and regenerative capabilities of transplanted stem cells has attracted great attentions. Currently, a series of NIR fluorescence imaging techniques have been developed for in vivo tracking of the transplanted stem cells.^[^ 93, 94, 95 ^]^

#### 2.2.1 Monitoring the Apoptosis or Death of Stem Cells

**Figure 4:**
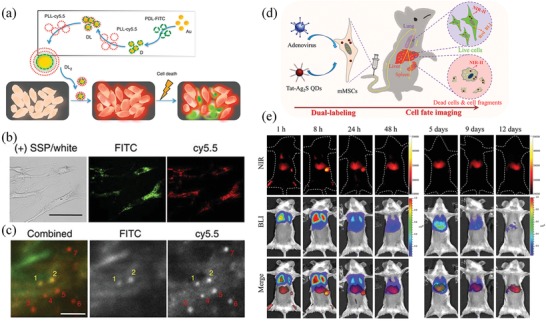
NIR imaging techniques for in vivo stem cell fate imaging. a) The synthesis of a chameleon‐like multilayered probe (DL2) for live‐ and dead‐cell imaging. b) In vitro imaging of apoptotic hMSCs by DL2. c) In vivo live/dead imaging of hMSCs injected in the dermis of the mouse ear pinna. Red: live cells. Yellow: apoptotic cells. Reproduced with permission.^[^ 69 ^]^ Copyright 2014, Nature Publishing Group. d) A dual‐modal BLI/NIRFI imaging technique for tracking the fate of transplanted stem cells in vivo. e) In vivo imaging the fate of intravenously transplanted MSCs in a mouse model of acute liver failure. NIR: NIR‐II fluorescence of Ag_2_S QDs. BLI: BLI signals of RFLuc. Reproduced with permission.^[^ 70 ^]^ Copyright 2018, Wiley‐VCH.

Recently, in vivo monitor of cell apoptosis or death of the transplanted stem cells has been achieved by using NIR imaging. In 2013, Lee et al. developed a chameleon‐like multilayered probe (named DL2) by assembling poly‐*d*‐lysine (PDL) with fluorescein isothiocyanate (FITC) and poly‐*l*‐lysine (PLL) with Cy5.5 on an AuNP.^[^ 69 ^]^ In the intact probe, the fluorescence of both Cy5.5 and FITC were quenched by the AuNP. After being taken up by living cells, the PLL could be degraded by intracellular proteases and the Cy5.5 was released to produce red fluorescence. During cell apoptosis, the protease‐resistant PDL could be degraded by the abnormally enhanced intracellular ROS and the FITC was released to produce green fluorescence (**Figure** **4**a). By this way, live cells could be distinguished from apoptotic and necrotic cells in real time. Thus, the fate of human mesenchymal stem cells (hMSCs) transplanted in the dermis of the mouse ear pinna was clearly observed in vivo by using the noninvasive NIR imaging method (Figure 4b,c). More recently, Chen et al. developed a dual‐modal BLI/NIR fluorescence imaging (NIRFI) strategy to monitor the fate of transplanted stem cells in vivo.^[^ 70 ^]^ In this study, MSCs were dual‐labeled by the NIR‐II emitting Ag_2_S QDs (1200 nm) and endogenous red‐emitting firefly luciferase (RFLuc, 620 nm) reporter gene (Figure 4d). Due to the high spatiotemporal resolution and deep tissue penetration of NIR‐II imaging, the NIR‐II fluorescence of Ag_2_S quantum dots could be applied to report the dynamic location of all transplanted MSCs. On the other hand, the BLI of RFLuc could be applied to specifically identify the living cells. By merging NIR‐II signals and BLI signals in the same mice, the living cells could be distinguished from dead cells in situ, because the living cells have both NIRFI signals and BLI signals while the dead cells have sole NIRFI signal. By utilizing the novel dual‐modal BLI/NIRFI imaging, the fate of the transplanted stem cells was clearly revealed in a mouse model of acute liver failure (Figure 4e). These promising cell fate imaging strategies may have great potential for evaluating the efficacy and safety of stem cell‐based therapeutics in vivo.

#### 2.2.2 Sensing the Signal Pathway and Differentiation of Stem Cells

Cell signals in terms of gene expression control a wide variety of crucial physiological processes in cells, including cell survival, proliferation, differentiation, and immunity.^[^ 96 ^]^ Thus, imaging of the cell signals has risen great attentions among researchers. Recently, several advanced NIR imaging techniques have been developed to in vivo sensing cell signaling in terms of monitoring the intracellular gene expressions, including endogenous reporter gene‐based fluorescence imaging and exogenous fluorescent probe‐based fluorescence imaging.^[^ 32, 36, 71, 72 ^]^

Currently, the endogenous reporter gene‐based fluorescence imaging is the most effective and popular cell signal imaging method. Because the reporter‐gene can be controlled under specific promoters, thus reporting specific gene expression. On the other hand, reporter proteins can be fused with target proteins, thus reporting the expression of target proteins. At present, the NIR fluorescent proteins engineered from pytochromes that generally have a peak emission from 669 to 720 nm were the most widely used genetically encoded NIR reporters, which have been introduced by several excellent reviews.^[^ 34 ^]^ In 2016, Shcherbakova et al. reported three bright monomeric NIR fluorescent proteins engineered from bacterial phytochrome for multiscale imaging cell signals. In their study, miRFP670, miRFP703 and miRFP709 were developed with a peak emission of 670, 703, and 709 nm, respectively. After being fused to specific target proteins, the developed NIR fluorescent proteins were successfully used as the cell cycle biosensor for detection of proliferation status of cells and the IkBa reporter for NF‐κB pathway in cultured cells and in animals.^[^ 36 ^]^

**Figure 5:**
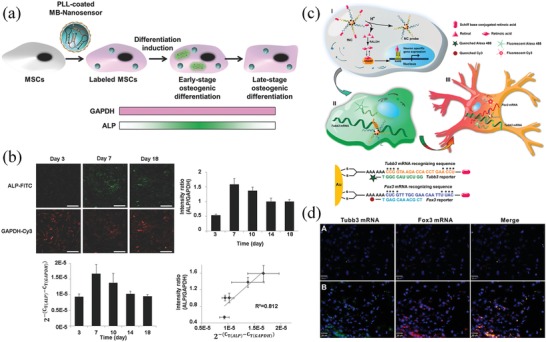
Exogenous NIR probes for sensing target gene expression in stem cells. a) A dual‐colored nanosensor for noninvasive monitoring of osteogenic differentiation of MSCs. b) Monitoring the early osteogenic differentiation of MSCs using the dual‐colored nanosensor. Reproduced with permission.^[^ 71 ^]^ Copyright 2016, Wiley‐VCH. The MB intensity (ALP/GAPDH) at different time points and the corresponding mRNA expression (ALP/GAPDH) from RT‐qPCR has a good linear relationship. c) A multifunctional nanocomplex for imaging of the Tubb3 and Fox3 mRNA expression during the process of neural stem cell differentiation. d) Imaging the spatiotemporal expression of specific mRNA in endogenous neural stem cells after stereotactical injection of the multifunctional nanocomplex into the lateral ventricle of the mouse brain. A: Mouse brain treated by nanocomplexes without retinoic acid. B: Mouse brain treated by nanocomplexes with retinoic acid. Green: Tubb3, red: Fox3, blue: Nucleus. Reproduced with permission.^[^ 72 ^]^ Copyright 2014, American Chemical Society.

At present, the available NIR fluorescent proteins are still limited and the maximal emission wavelength of the available NIR fluorescent proteins was generally shorter than 800 nm. Currently, a series of exogenous NIR nanoprobes have also been developed to monitor the cell signals in living cells and organisms. In 2016, Wiraja and colleagues reported a dual‐colored nanosensor for noninvasive monitoring of the osteogenic differentiation of MSCs.^[^ 71 ^]^ In this study, two molecular beacon (MB) probes, ALP‐FITC and glyceraldehyde‐3‐phosphate dehydrogenase (GAPDH)‐Cy3, were encapsulated within the biodegradable poly (lactic‐*co*‐glycolic) (PLGA) polymeric nanoparticles. Upon internalization in cells, the ALP‐FITC and GAPDH‐Cy3 MBs reacted with their complementary target oligonucleotides and turned on the fluorescence of FITC and Cy3, thus reporting the mRNA of ALP and GAPDH in cells. ALP is an early osteogenic differentiation marker that is overexpressed in the early stage of osteogenic differentiation. Meanwhile, GAPDH is a housekeeping gene that is stably expressed during osteogenic differentiation and can be used as an internal reference. Therefore, the osteogenic differentiation of MSCs could be dynamically monitored by normalizing the ALP‐MB signal with the corresponding GAPDH‐MB signal (**Figure** **5**a,b). In another example, Wang et al. synthesized a multifunctional nanocomplex that was capable of imaging the spatiotemporal expression of specific mRNA during the differentiation of neural stem cells without the use of transgenetic manipulation.^[^ 72 ^]^ In the intact nanocomplex, DNA oligonucleotides with an Alexa488 tag or Cy3 tag were conjugated on the Au nanoparticles. Thus, the fluorescence of both Alexa488 and Cy3 tags were quenched in the intact nanocomplex. Upon reaction with the target Tubb3 or Fox3 mRNA that expressed at specific differentiation stages of NSCs, the responding oligonucleotides with Alexa488 or Cy3 tag were released and produced green or NIR fluorescence, respectively. In this way, it was able to monitor the Tubb3 or Fox3 mRNA activities in both the cultured NSCs and endogenous NSCs at specific differentiation stages. By amending corresponding oligonucleotide sequences in the nanocomplex, this strategy also can be further used as an amenable tool to explore the dynamics of intricate mRNA activities in various sophisticated biological events, such as cancer progression and embryo development (Figure 5c,d).

In a more recent study by Wang's group, the endogenous reporter gene‐based imaging and exogenous fluorescent probe‐based fluorescence imaging were integrated to fully understand the fate of the transplanted stem cells including distribution, viability, and differentiation activities of transplanted stem cells.^[^ 32 ^]^ First, the Ag_2_S QD‐based NIR‐II imaging was applied to monitor the translocation of transplanted hMSCs due to the high spatiotemporal resolution and high tissue penetration of NIR‐II imaging. Second, the CMV promoter‐driven RFLuc‐based BLI was used to monitor the viability of transplanted hMSCs because both the expression of CMV promoter‐driven RFLuc and the BLI intensity was highly dependent on the viability of cells. Third, the collagen type 1 (col1α1) promoter‐driven Gaussia luciferase (GLuc)‐based BLI were used to monitor the osteogenic differentiation statuses of hMSCs because the col1α1 promoter was highly active during the early stage of osteogenic differentiation of stem cells. By integrating all the three fluorescence signals in the same mouse, the distribution, viability, and osteogenic differentiation of transplanted hMSCs were simultaneously visualized in a calvarial defect mouse model. This novel multiplexed imaging strategy offers a powerful tool to in vivo report multiple functions of stem cells, which will have great potential for a series of biomedical applications, such as therapeutic efficacy evaluation and imaging‐guided cell therapeutics.

### 2.3 NIR Imaging for Neural Activity Sensing in Neuroscience

Membrane electrical activity of cells involved in a variety of cellular processes such as cell signaling, cell–cell communication, and ion conductivity.^[^ 97, 98 ^]^ In particular, the membrane electrical activity of neural cells controls the basic functions of the brain. Thus, monitoring the electrical activity of neural cells is of great importance for evaluating how the nervous system works and how neurodegenerative diseases develop. Therefore, the development of noninvasive techniques for monitoring the membrane potential of cells has attracted great attentions in neuroscience research.^[^ 99, 100, 101 ^]^

Due to the high spatiotemporal resolution and wide imaging field of view, fluorescence imaging is capable of simultaneously monitoring a large number of neurons with subcellular resolution, which is unachievable by using electrode‐based measurements.^[^ 102 ^]^ Thus, fluorescence imaging method has become one of the most important methods in neural activity sensing. Currently, a series of fluorescent voltage indicators, such as genetically encoded sensors, small molecule fluorescent voltage sensors, genetically encoded protein‐dye hybrid indicators and QD‐based voltage sensors, have been developed for imaging neural activity.^[^ 100, 101, 103, 104 ^]^ In particular, the development of voltage‐sensitive NIR probes have offered a possibility of imaging the neural activity in deep tissues with ideal spatiotemporal resolution.

**Figure 6:**
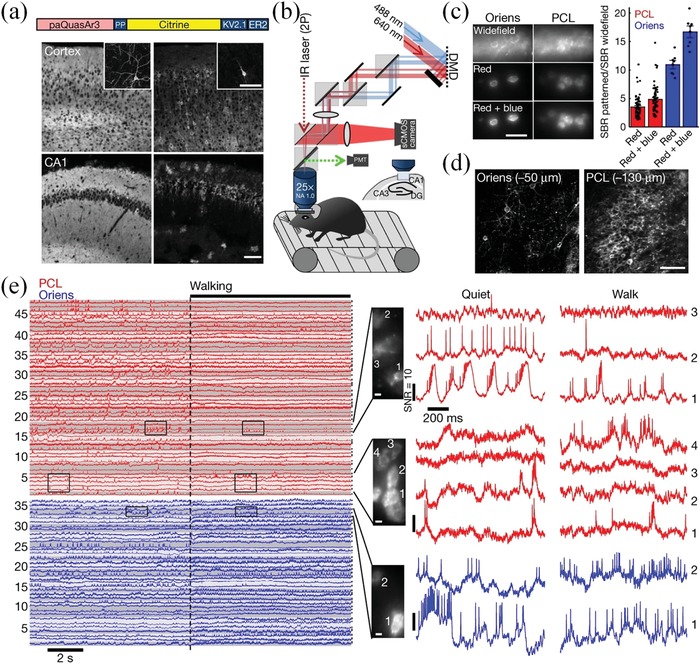
Optical imaging of neuronal activity in hippocampus of mice using paQuasAr3‐s. a) Construction of paQuasAr3‐s and confocal images of brain slices expressed paQuasAr3 and paQuasAr3‐s. Scale bars, 100 µm. b) Scheme of the optical system for simultaneous two‐photon (2P) imaging and patterned illumination with red and blue light. c) Epifluorescence images (Left) and quantification (Right) of paQuasAr3‐s expression in the CA1 region of the hippocampus. Scale bar, 50 µm. d) paQuasAr3‐s expression in the Oriens (left) and the PCL (right) imaged by two‐photon fluorescence imaging. Scale bar, 100 µm. e) Fluorescence recordings of neuronal activity from PCL (red) and Oriens (blue, *n* = 5 mice). Reproduced with permission.^[^ 73 ^]^ Copyright 2019, Nature Publishing Group.

Nowadays, a series of genetically encoded voltage sensitive proteins have been developed by using different voltage‐response principles, such as the voltage‐sensitive phosphatases (VSPs)‐based indicators and the rhodopsin‐based indicators.^[^ 105, 106 ^]^ The VSPs‐based indicators generally contain VSPs and fluorescent proteins (FPs).^[^ 107 ^]^ The conformational change in voltage sensitive domain after depolarization cause change in fluorescence intensity of FPs, thus reporting the change of membrane potential in cells. For the rhodopsin‐based indicators, voltage‐dependent change in photophysical state of opsin can alter the fluorescence intensity of spectrally compatible FPs via FRET, thus sensing the membrane potential of cells.^[^ 108, 109 ^]^ However, most of available genetically encoded voltage indicators (GEVIs) emit fluorescence at the visible window, which limiting their applications in deep tissues in living systems. In 2019, Cohen's group developed a genetically encoded NIR voltage indicator (named QuasAr3) with a maximal emission wavelength of 720 nm (**Figure** **6**).^[^ 73 ^]^ In this system, the QuasAr3 was illuminated in a voltage‐dependent manner under continuous excitation (λ_ex_ = 640 nm, 10 W mm^−2^). Moreover, the NIR fluorescence (λ_em_ = 660–740 nm) of QuasAr3 was reversibly activated by a moderate‐intensity blue light (λ_act_ = 488 nm, 100 mW mm^−2^). For post‐synaptic potentials (PSPs), a high voltage sensitivity (Δ*F*/*F* = 40 ± 1.7% per 100 mV) was achieved. Thus, the supra‐ and subthreshold voltage dynamics in multiple neurons were real‐time recorded in the hippocampus of behaving mice (Figure 6e). Moreover, the NIR voltage sensing system was able to be integrated light stimulation using optogenetics, thus enabling detailed exploring of network dynamics in the context of behavior.

Another type of most widely used voltage‐sensitive probe is the small molecule dye. In the early 1970s, Cohen and colleagues screened thousands of dyes for compounds with voltage‐sensitive optical properties. Merocyanine 540, one of the first voltage‐sensitive fluorescent dyes, was found to be able to display large squid axon action potential.^[^ 110 ^]^ After that, many voltage‐sensitive small molecules were discovered or synthesized, including di‐4‐ANEPPS, ANNINE6, ICG, and BeRST1.^[^ 39, 111 ^]^ These small molecule dyes sense membrane potentials of cells generally via a mechanism of electrochromic based on the stark effect or photoinduced electron transfer.

**Figure 7:**
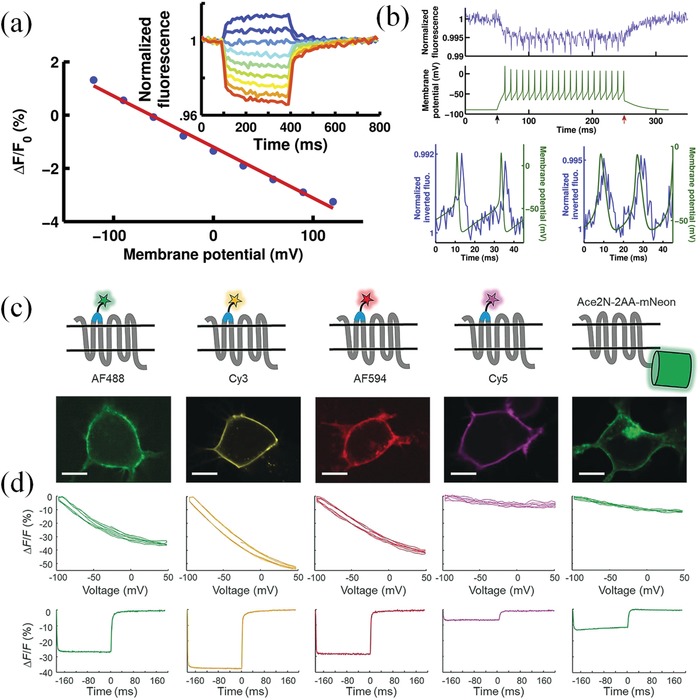
NIR organic dyes and NIR dye‐membrane protein hybrid probes for membrane potential imaging. a) The fluorescence intensity of ICG (blue points) is roughly linearly related (red line, fit to data) with voltage in the ICG‐labeled oocytes. Ex: 780 nm, Em: 818–873 nm. b) Action potential monitor by ICG in the ICG‐labeled oocytes. The fluorescence of ICG (blue) were detected at a rate of 107 Hz. The corresponding action potentials (green) were recorded under current clamp. Reproduced with permission.^[^ 74 ^]^ Copyright 2014, Elsevier Inc. c) The design of microbial rhodopsin protein and organic fluorophore hybrid (FlareFRET) sensors and the confocal images of HEK cells labeled by different FlareFRET sensors and Ace2N‐2AA‐mNeon. Scale bars: 10 µm. d) Fluorescence as a function of membrane voltage and fluorescence response of different FlareFRET sensors and Ace2N‐2AA‐mNeon to a stepping membrane voltage between −70 mV and 30 mV. Reproduced with permission.^[^ 76 ^]^ Copyright 2018, Wiley‐VCH.

Recently, lots of efforts have been paid to develop NIR voltage‐sensitive dyes. In 2012, Loew's group synthesized a series of fluorinated styryl dyes with emission wavelength between 440 and 670 nm.^[^ 112 ^]^ In 2014, Bezanilla and colleagues found that the US Food and Drug Administration (FDA)‐approved ICG (Ex: 780 nm, Em: 818–873 nm) could be served as a voltage‐sensitive indicator.^[^ 74 ^]^ It was found that the fluorescence change of ICG was roughly linearly related to the change of membrane potential and had a magnitude of ≈1.9% of the baseline fluorescence per 100 mV of membrane potential change (**Figure** **7**a). Additionally, ICG has a high time response with a primary time constant of 4 ms, which contributes to the successful monitoring of the action potential (Figure 7b). Moreover, the repetitive firing and hyperexcitability were clearly distinguished by ICG fluorescence even when the stimulation was stopped. In addition, frog and rat cell experiments have shown that ICG voltage response characteristics are applicable not only to specific animals or cell lineages, but also to a wide range of tissues. For complex tissues such as hippocampal sections of rats, brain excitation signals generated by electric field electrode stimulation could be well reflected by ICG fluorescence changes. In 2016, Martisiene and colleagues promoted ICG to monitor cardiac electrical activity.^[^ 75 ^]^ They found that ICG had a fast and slow dual‐component response to membrane potential changes. Two different voltage‐sensitivity mechanisms for ICG were revealed by separating the optical signal into the two components. First, the fast component of the optical signal may attribute to the electrochromic nature of ICG. Second, the slow component may arise from the redistribution of ICG within or around the membrane.

Genetically encoded voltage indicators are readily targetable to specific cell types and are able to monitor neuronal activity at high spatiotemporal resolution. However, the brightness and photostability of available fluorescent proteins and rhodopsins are typically worse than small molecule dyes. Thus, the hybrid voltage indicators have attracted great attentions among researchers due to their unique advantages to combine the merits of genetically encoded proteins and small molecule dyes.^[^ 113, 114, 115 ^]^ For example, Abdelfattah and colleagues recently developed a hybrid voltage indicator by combining the rhodopsin and small molecule JF525 (Voltron525).^[^ 113 ^]^ In this study, the HaloTag was fused to the rhodopsin. JF525 was targeted to the rhodopsin due to the specific binding affinity between JF525 and the HaloTag. Therefore, a high voltage sensitivity (−23 ± 1% Δ*F*/*F*0 per 100 mV) was achieved by using the hybrid indicator. Moreover, this indicator worked in a wide range of cells and tissues and had been successfully applied to sense action potentials in the mouse, zebrafish, drosophila neurons.

In another study, a series of voltage sensitive indicators (FlareFRET) were developed by the site‐specific modification of microbial rhodopsin proteins with different organic fluorophores, including AF488, Cy3, AF594, and Cy5 (Figure 7c,d).^[^ 76 ^]^ Because membrane voltage can alter the absorption spectrum of rhodopsin via modulating the acid‐base equilibrium of a Schiff base. The fluorescence of FlareFRET could be altered according to the membrane voltage via the electrochromic fluorescence resonance energy transfer (eFRET) between rhodopsin and organic dyes. To correctly bind dyes in a specific site of the rhodopsin, the authors fused a 13‐amino‐acid acceptor peptide (LAP) to rhodopsin and ligated a picolyl azide (pAz) moiety to its lysine side chain. Thereby, the alkyne‐derivatized fluorophore could effectively conjugated to pAz via Cu‐catalyzed azide‐alkyne cycloaddition (CuAAC) reaction. After inserting LAP at various locations in the Ace sequence, they found the Flare1 indicator with a Cy3 conjugation in the first extracellular loop of Ace was the most sensitive and fastest voltage indicator with a Δ*F*/*F*0 of −35.9% fluorescence changes per 100 mV. The high sensitivity of Flare1 provided a good solution to establish an optical mapping of electrical connections between cultured cells.

In recent years, the application of fluorescent QDs for monitoring electrical activity of cells has also attracted great attentions due to the excellent optical properties of QDs, such as photobleaching resistance, high fluorescent yield and easy functionalization.^[^ 104 ^]^ In 2019, Wang's group developed a FRET‐based voltage imaging system by using the CdSe@ZnS‐GSH QDs and the dipicrylamine (DPA), showing excellent voltage‐dependent fluorescence intensity and optical stability during imaging.^[^ 116 ^]^ In 2017, Nag and colleagues developed a QD‐peptide‐fullerene bioconjugation for cellular membrane potential imaging.^[^ 77 ^]^ The nanobioconjugate comprised an electron donor (QD: CdSe/CdS/ZnS, Ex = 605 nm) and an electron acceptor (fullerene). The electron transfer‐driven process between electron donor and electron acceptor made the fluorescence intensity of the nanobioconjugate change steadily according to the changes of membrane potential. A Δ*F*/*F*0 of 2–20% depending on the QD‐C60 separation distance was observed. The nanobioconjugate features strong photostability and a large two‐photon cross section (usually 10^3^–10^4^ times the size of organic dyes), allowing voltage sensitive probes for deep tissue imaging. However, the QD‐based voltage indicator with a wavelength of longer than 700 nm has not been developed yet. Thus, further study by using NIR QDs with longer wavelength is needed to promote the application of QD‐based voltage probes in neuroscience research.

## 3 Advanced NIR Light for Spatiotemporal Modulation of Cell Functions

The spatial and temporal modulation of cells has generated great interest among researchers for their potential applications in numerous fields. Currently, light has been one of the most widely used tools for its feasibility in cell control and modulation with exceptional spatiotemporal precision and sensitivity.^[^ 6, 42, 43 ^]^ In particular, NIR light is capable of penetrating into the deep tissues thus can be served as a noninvasive wireless tool to control specific cells in deep tissues in vivo. Currently, a series of NIR light‐responsive nanomaterials have been developed for photoregulation in biomedicine, such as UCNPs, AuNPs, and organic semiconducting polymers.^[^ 5, 26, 42, 48, 117 ^]^ Herein, the latest research progress of the development of novel photoregulation strategies and their applications in cancer therapy, regenerative medicine, and neuroscience were summarized and discussed (**Table** **2**).

**Table 2:** Representative researches of NIR photoregulation in living system

Research field	Photosensitive materials	Light wavelength [nm]	Mechanisms	Applications	Reference
Cancer therapy	Flav7‐based macromolecular fluorophore	808	Photothermal therapy	In vivo carcinoma tumor treatment	^[^ 118 ^]^
	Semiconducting polymer nanoblockader (SPN_B_)	808	Photodynamic therapy	In vivo xenografted 4T1 tumor treatment	^[^ 26 ^]^
	UCNP‐(CD/Azo)‐siRNA/PEG NPs	980	Release of siRNA by regulating Azo‐CD interaction	Gene silencing in MDA‐MB‐468 TNBC cells	^[^ 119 ^]^
	UCNPs & Opto‐CRAC	980	Regulation Ca^2+^‐responsive genes	In vivo trigger immune responses and kill tumor cells	^[^ 120 ^]^
	UCNPs & Cry2‐Cib1	980	Light‐activated Cry2‐Cib1 interaction	Control apoptotic signaling pathways in cancer cells	^[^ 121 ^]^
	Hollow gold nanoshells (HGNs) & Cre recombinase	808	Activating Cre recombinase by the photothermal effects of HGNs	Control gene recombination in HeLa cells	^[^ 122 ^]^
	Semiconductor polymer brush (SPPF) & CRISPR/Cas9	808	Photothermal conversion of SPPF	MTH1 gene editing in HCT 116‐GFP tumor model	^[^ 123 ^]^
	Semiconducting polymer nanotransducer (pSPN) & CRISPR/Cas9	680 or 808	Generated ^1^O_2_ to cleave the ^1^O_2_‐cleavable linkers and release the CRISPR/Cas9 plasmids	Gene editing in Hela cells	^[^ 124 ^]^
	UCNPs & CRISPR/Cas9	980	Cleavage of photosensitive molecules by UCNP generated UV light	In vivo inhibition of the PLK‐1 gene	^[^ 125 ^]^
	Black phosphorous (BP) nanosheets & agarose hydrogel	808	Control of drug release through the photothermal effects of BP	Treatment of MDA‐MB‐231 tumors	^[^ 126 ^]^
Regenerative medicine	Upconversion nanotransducers	980	Control the release of caged molecules via light‐controlled ONA‐β‐CD interaction	Control of hMSCs differentiation into chondrocytes, hypertrophic chondrocytes or osteoblasts	^[^ 127 ^]^
	Mesoporous silica‐coated UCNPs	980	Photocontrolled siRNA delivery	Promote osteogenic differentiation of hMSCs	^[^ 128 ^]^
	Gold nanorods (AuNRs) & DNA agonist	808	Control of DNA agonist release through the photothermal effects of AuNRs	Activation of the RTK signal in cells for muscle regeneration	^[^ 27 ^]^
	Polyelectrolyte multilayer microcapsule	830	Control of ALP release through the photothermal effects of Au NPs	Manipulate the Wnt/β‐catenin signaling pathway	^[^ 129 ^]^
	Gold nanoshells (NSs)	808	Directly stimulate heat shock proteins and sirtuin 1 genes	Myotube activation	^[^ 130 ^]^
	Light‐sensitive BphS & CRISPR‐dCas9	730	Up‐regulated the NEUROG2 by NIR light	Promote differentiation of induced pluripotent stem cells into neurons	^[^ 131 ^]^
	Bismuth sulfide/hydroxyapatite (BS/HAp) film	808	NIR light‐induced electron transfer	Promote osteogenic differentiation and bone regeneration	^[^ 54 ^]^
	UCNP/PAAm/HA‐RB	980	NIR light‐induced cross‐linking of collagens	Wound regeneration	^[^ 55 ^]^
Neuroscience	IlaM5	730	A Bph‐based optogenetic system	Control the brain activity of living mice	^[^ 132 ^]^
	UCNPs & ReaChR	800	Activate ReaChR with NIR light	Control the activity of hippocampal neurons	^[^ 133 ^]^
	Uranium‐doped UCNPs & Chrimson	808	Activate Chrimson and inhibit GABAergic motor neurons	Control the movement behavior of Caenorhabditis elegan	^[^ 134 ^]^
	Blue‐emitting UCNPs & rhodopsin	980	Active the channel rhodopsin	In vivo activate or inhibit the activity of neurons in the deep brain	^[^ 28 ^]^
	pbUCNPs	980	Transform invisible NIR light into visible emissions	Help mammalian to acquire NIR light image vision	^[^ 135 ^]^
	Silica‐coated gold nanorods (Au NRs)	780	Photothermal effects of Au NRs	Stimulate the activity of primary auditory neurons	^[^ 52 ^]^
	Semiconducting polymer nanobioconjugates (SPNsbc)	808	Photothermal effects of SPNsbc	Control the thermosensitive TRPV1 ion channels in neurons	^[^ 136 ^]^

### 3.1 NIR Light for Cancer Therapy

In recent years, NIR light‐based tumor therapy has received numerous attentions due to its potential applications in precision medicine. At present, a series of light‐based strategies have been developed for cancer treatment, including photothermal therapy (PTT), PDT, light‐based gene therapy, and light‐controlled drug delivery and release.^[^ 5, 16, 42 ^]^

#### 3.1.1 Photothermal Therapy and Photodynamic Therapy for Cancer

**Figure 8:**
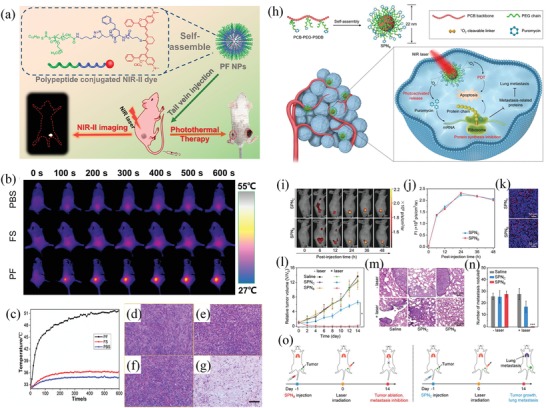
Photothermal therapy and photodynamic therapy for cancer. a) Scheme of the NIR image‐guided photothermal therapy by a facile macromolecular fluorophore. b) IR thermal images of the mice after intravenous injection with PBS, Flav7, and macromolecular fluorophore under laser irradiation. c) Temperature profile of the tumors. d–g) H&E images of tumors collected from different groups (scale bar 100 µm). Reproduced with permission.^[^ 118 ^]^ Copyright 2019, American Chemical Society. h) A NIR photoactivatable semiconducting polymer nanoblockader (SPN_B_) for photodynamic therapy. i) NIR fluorescence images of 4T1‐tumor‐bearing mice with SPN_B_ or SPN_C_ treatment. j) NIR fluorescence intensities of tumors at different time points. k) Fluorescence images of tumor tissues treated with SPN_B_ or SPN_C_. Blue: cell nucleus stained with DAPI; red: nanoparticles. l) Growth curves of tumors in 4T1‐tumor‐bearing mice with different treatments. m) H&E staining images of lung metastases. n) Numbers of metastasis nodules in lung from mice with different treatments. o) Scheme of SPN_B_‐mediated cancer therapy. Reproduced with permission.^[^ 26 ^]^ Copyright 2019, Wiley‐VCH.

PTT is a noninvasive and remote‐controllable strategy for cancer treatment. For PTT, photothermal agents are generally used to convert external NIR light into local heat and ablate tumors. Nowadays, PTT has been widely used for tumor treatment by using various photothermal agents, such as Au nanomaterials, organic dyes and polymer semiconductors.^[^ 50, 51, 66, 67, 118 ^]^ In 2019, Yan and Wang et al. developed a macromolecular fluorophore (PF) nanoparticle using an amphiphilic polypeptide‐conjugated NIR‐II fluorophore (Flav7) (**Figure** **8**a–g).^[^ 118 ^]^ The synthesized PF nanoparticles showed satisfactory photothermal conversion efficiency (42.3%) and excellent photothermal stability. Moreover, the accumulation of PF nanoparticles in tumors could be monitored by the NIR‐II fluorescence of PF nanoparticles, thus improving the diagnosis accuracy and treatment outcomes. In a murine mammary carcinoma tumor model, the PF‐based PTT successfully ablated the tumors. In combination with the inherent biodegradability and biocompatibility of PF nanoparticles, the PF‐based PTT strategy may be a promising strategy for accurate diagnosis and remote‐controllable PTT of tumor.

In addition to PTT, PDT is also widely used for cancer therapy. For PDT, photosensitive agents can generate ROS (singlet oxygen) under NIR irradiation, thus killing cancer cells by PDT. In recent years, a series of agents have been developed for PDT, such as semiconducting polymer nanoparticles.^[^ 26, 137 ^]^ For example, Pu and colleagues synthesized a semiconducting polymer nanoblockader (SPN_B_) for metastasis‐inhibited cancer therapy (Figure 8h).^[^ 26 ^]^ In this study, an amphiphilic semiconducting polymer was grafted with a protein biosynthesis blockader‐conjugated poly(ethylene glycol) to generate SPN_B_. The SPN_B_ was able to generate singlet oxygen (^1^O_2_) under NIR photoirradiation, thus killing tumor cells via PDT. In addition, the protein biosynthesis blockader could be released after the break of ^1^O_2_ cleavable linker by the generated ^1^O_2_ to terminate protein translation in cancer cells. In a xenografted 4T1 tumor model, SPN_B_ showed a synergistic effect of PDT and inhibition of protein synthesis, offering a significantly enhanced therapeutic efficacy in tumor ablation (Figure 8i–o). In another example, Dong and colleagues developed FA‐CuS/DTX@PEI‐PpIXCpG nanocomposites for synergistic phototherapy (include PDT and PTT) and docetaxel (DTX)‐enhanced immunotherapy,^[^ 137 ^]^ which showed highly efficient for breast cancer treatment.

#### 3.1.2 NIR Light‐Based Gene Expression Regulation for Cancer Therapy

Light may be the most promising tool for precise control of spatiotemporal gene expression in multicellular organisms because it is easy to obtain, highly tunable, non‐toxic, and most importantly, has high temporal and spatial resolution.^[^ 42 ^]^ The introduction of NIR light allows to accurately determine when and where the activation or inhibition of the specific gene expression happened. Currently, NIR‐light‐mediated regulations of gene expression were mainly achieved through NIR light nanotransducers, such as upconversion nanoparticles (UCNPs), gold nanorods (AuNRs), and photothermal polymers.^[^ 5, 42, 48, 49 ^]^

UCNPs can convert tissue‐permeable NIR light into UV or visible light. Subsequently, the UV or visible light can be utilized to control the optogenetic channel proteins and photosensitive polymers, thus regulating the gene expression of target cells.^[^ 48 ^]^ In recent years, UCNP‐mediated optogenetics has been widely reported. For example, Chen and colleagues reported a UCNP‐based siRNA nanocarrier system for spatiotemporally control of gene silencing in MDA‐MB‐468 TNBC cells.^[^ 119 ^]^ In this study, siRNA was conjugated to the NaYF_4_:Yb/Tm/Er UCNP via an azobenzene (Azo)‐cyclodextrin (CD) host–guest interaction. The UCNP converted the tissue‐permeable NIR light into UV light and the generated UV emission triggered the trans‐to‐cis photoisomerization of Azo to release the siRNA, achieving spatiotemporally control of gene silencing.

**Figure 9:**
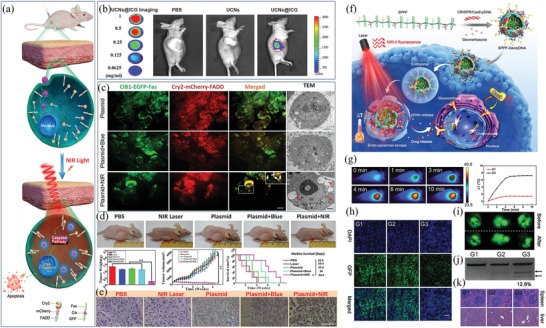
Photoregulation of gene expression for cancer therapy. a) NIR‐controlled upconversion optogenetic nanosystem for tumor suppression. b) In vitro and in vivo imaging of UCNs@ICG with various concentrations. c) Confocal and TEM images of tumor sections after different treatments. Green: EGFP; Red: mCherry. d) Therapeutic efficacy of different treatments for tumor after 4 weeks. e) TUNEL staining of tumors with different treatments. Reproduced with permission.^[^ 121 ^]^ Copyright 2017, American Chemical Society. f) Schematic illustration of SPPF‐Dex nanoparticles for CRISPR/Cas9 delivery and NIR light‐controlled genome editing. g) IR thermal images of tumor‐bearing mice under laser irradiation (808 nm, 0.45 W cm^−2^). h) Confocal images of tumor sections with different treatment. Green: GFP; Blue: Hoechst 33342‐stained nuclei. Scale bar = 50 µm. i) Fluorescence images of tumors before and after treatment. j) T7E1 assays of PCR products of GFP gene from tumors with different treatment. k) H&E images of spleen and liver sections. G1: tumor treated with PBS and 808 nm laser; G2: tumor treated with SPPF‐Dex/Cas9‐sgGFP NPs; G3: tumor treated with SPPF‐Dex/Cas9‐sgGFP NPs and 808 nm laser. Reproduced with permission.^[^ 123 ^]^ Copyright 2019, Wiley‐VCH.

Recently, photoregulation of the cell signaling in immune cells has also been developed for cancer therapy. In 2017, Han's group constructed a NIR‐stimulated optogenetics platform (named “Opto‐CRAC”).^[^ 120 ^]^ In this platform, UCNP was used as a nanotransducer to convert tissue‐permeable NIR light into visible emission. Then, the generated visible light selectively and remotely controlled the expression of Ca^2+^ oscillations and Ca^2+^‐responsive genes in immune cells, such as T lymphocytes, macrophages and dendritic cells. By using this platform, the Ca^2+^‐dependent master transcriptional regulator NFAT (nuclear factor of activated T cells) were triggered to subsequently cause NFAT nuclear translocation in cells. In a melanoma mouse model, Opto‐CRAC was shown to act as a “photoactivated adjuvant” encoded by the gene, which improved antigen‐specific immune responses and specifically destroyed tumor cells. In another example, Chang et al. designed an upconversion optogenetic nanosystem by combining UCNPs and the blue light‐mediated heterodimerization modules (**Figure** **9**a–e).^[^ 121 ^]^ In this system, UCNPs converted external NIR light to local blue light to noninvasively activate photoreceptors. Then, the blue light‐activated arabidopsis flavoprotein cryptochrome 2 (Cry2) quickly interacted with its partner Cib1 and recruited FADD to Fas on the plasma membrane, therefore, activated the caspase pathway in cells. Due to the deep penetration depth of NIR light, it was demonstrated that this optogenetic nanosystem could be used to control apoptotic‐signaling pathways in mammalian cells and cancer cells in mice. In a mouse model with HeLa xenograft tumor, the NIR‐triggered optogenetic strategy successfully induced cancer cell apoptosis and inhibited the growth of tumors in vivo (Figure 9d,e).

Photoregulation of gene‐editing proteins (such as Cre and CRISPR/Cas) to cut or paste specific parts of a target DNA sequence has also received vast attention in cancer therapy. Photoactivated Cre recombinase (PA‐Cre) is widely used as an optogenetic tool for stably modifying gene expression in cells.^[^ 122, 138 ^]^ In 2018, Reich and coworkers proposed a NIR photoactivated Cre recombinase genome editing platform for gene recombination in HeLa cells.^[^ 122 ^]^ In this platform, a TAT peptide fusion of Cre recombinase was assembled on the plasma hollow gold nanoshells (HGNs) via the interaction between polyhistidine tags and divalent cations. Upon NIR irradiation (808 nm), the Cre recombinase was released and high‐throughput photoactivated genome editing was achieved. With such light‐responsive properties, this novel strategy has great prospects for controlling gene expression in cell therapy. In addition to Cre, the light‐controlled CRISPR‐associated protein 9 (Cas9) genome‐editing system has also been developed and shown great potential in cancer therapy. In 2019, Chen and colleagues designed a semiconductor polymer brush (SPPF) to remote control of CRISPR/Cas9 genome editing via NIR light (Figure 9f).^[^ 123 ^]^ In this system, SPPF was served not only as a carrier for delivering CRISPR/Cas9 boxes but also a controller for lysosomal escaping and payload releasing of CRISPR/Cas9 boxes through photothermal conversion under NIR light illumination. Moreover, the SPPF‐based NIR‐II imaging was also used to monitor the in vivo distribution of genome editing systems and direct laser irradiation in real time. In a HCT 116‐GFP tumor model, indel mutations of the MTH1 (MutT Homolog1) at a frequency of approximate 20% were achieved by using the SPPF‐Dex/Cas9‐sgMTH1 treatment along with laser irradiation (Figure 9g–k).

In addition to above mentioned photothermal‐based strategies, cleavable linker‐based strategies also have been used for CRISPR/Cas9 activation in cancer therapy. In 2019, Pu et al. developed a photolabile semiconducting polymer nanotransducer (pSPN) to deliver CRISPR/Cas9 plasmids and active gene editing in cells.^[^ 124 ^]^ The synthesized pSPN comprises a ^1^O_2_‐generating backbone, ^1^O_2_‐cleavable linkers and polyethylenimine brushes. Upon NIR photoirradiation (680 or 808 nm), the generated ^1^O_2_ cleaved the ^1^O_2_‐cleavable linkers, thereby releasing the CRISPR/Cas9 plasmids and initiating gene editing. By using this strategy, a 15‐ and 1.8‐fold enhancement in target gene expression relative to the controls in Hela cells and mice.

In another interesting study, Song and colleagues successfully developed a NIR light‐responsive CRISPR‐Cas9 nanocarrier for cancer therapeutics.^[^ 125 ^]^ They used UCNPs to generate UV light with a NIR excitation of 980 nm to cleave the photosensitive molecules and released the CRISPR‐Cas9 from the UCNP nanocarriers for gene editing. By using this method, the cancer cell proliferation and tumor growth were successfully inhibited by targeting the PLK‐1 gene (a marker of cancer gene). These exogenous NIR light‐controlled methods provided new avenues for precise gene therapy for cancer treatment in the near future.

#### 3.1.3 Light‐Responsive Hydrogels for Cancer Therapy

In addition to light‐responsive nanoparticles, NIR lightresponsive hydrogels have also been developed for cancer therapy through precise control of biomacromolecules (protein and enzyme) and drugs.^[^ 2, 126, 139 ^]^

**Figure 10:**
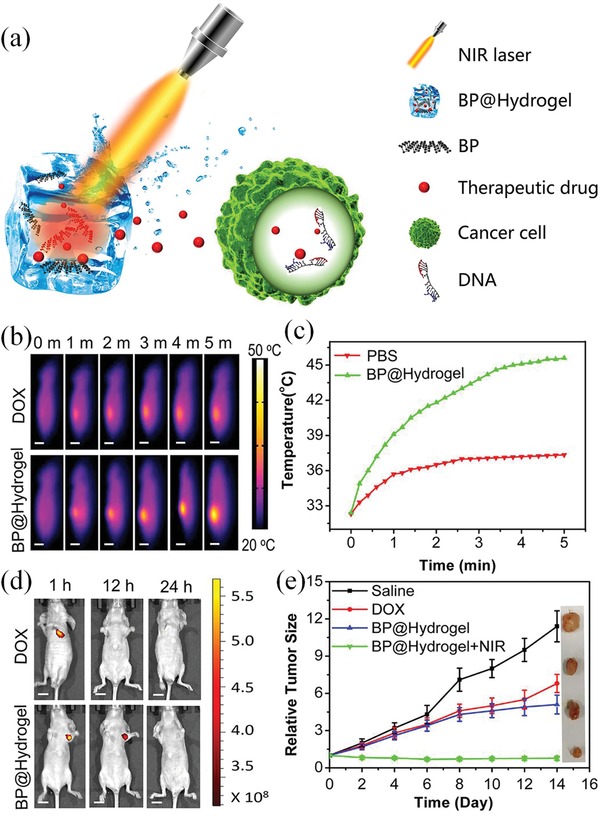
NIR light‐responsive BP@Hydrogel for cancer therapy. a) The working principle of BP@Hydrogel. Upon NIR‐light irradiation, BP@Hydrogel released the encapsulated chemotherapeutics to break the DNA chains and induced apoptosis in cells. b) Thermal images of tumor bearing mice after DOX or BP@Hydrogel treatment and 808 nm laser irradiation (1.0 W cm^−2^, 5 min). c) Tumor temperature changes of mice in (b). d) Fluorescence images of mice after in vivo photothermal assay. e) Growth curves of tumors in mice with different treatments. Reproduced with permission.^[^ 126 ^]^ Copyright 2018, PNAS.

In 2019, Cao and coworkers developed a photosensitive hydrogel that could control the release of various drugs by NIR light for cancer treatment.^[^ 126 ^]^ The authors encapsulated PEGylated black phosphorous (BP) nanosheets in a low‐melting‐point agarose hydrogel. The encapsulated BP nanosheets efficiently converted NIR light (λ_ex_ = 808 nm) into heat. Under the illumination of NIR light, the BP‐encapsulated hydrogel changed from a solid state to a molten state, thereby achieving the controlled release of DOX and hydrogel degradation. Moreover, the release rate of the drug can be precisely adjusted by regulating internal parameters (such as the concentrations of agarose, BP, and drug) and external parameters (such as light intensity, and exposure time) to achieve precise treatment of cancer, for example, breast and melanoma, with high efficiencies and negative side effects (**Figure** **10**). In another study, Zhao and coworkers created a light‐sensitive hybrid hydrogel by encapsulating NaYF_4_:TmYb core–shell UCNPs in the cross‐linked hybrid polyacrylamide‐poly(ethylene glycol) (PEG) hydrogel.^[^ 139 ^]^ The UCNPs induced a gel‐sol transition when they were irradiated with 980 nm NIR light. Since the UCNPs converted five NIR photons into a UV photon, the UV light was then emitted back into the system to trigger the photoresponsive *o*‐nitrobenzyl groups within the PEG hydrogel. As a result, the hydrogel was gradually degraded to release the entrapped biomacromolecules on demand. With the capability of precisely controlling drug releasing and activity, the NIR‐responsive hydrogel may serve as a promising tool for precision cancer therapy in the future.

### 3.2 NIR Light for Cell Fate Regulation in Regenerative Medicine

The efficacy of stem cell‐based therapy is primarily determined by their fate after transplantation, including migration, proliferation, paracrine and differentiation.^[^ 92, 140 ^]^ Remote control of stem cell fate in vivo by the noninvasive stimulation is of great appealing for regulating and enhancing stem cell‐based therapy. In particular, the NIR light‐based manipulation strategies are able to precisely control target cells in deep tissues with high spatial and temporal resolution, thus modulating the ways of transplanted stem cells work.^[^ 5, 42 ^]^

#### 3.2.1 NIR Light‐Based Gene Expression Regulation for Stem Cell Modulation

The fate of cells is often determined by complex cellular signaling pathways. Currently, a series of NIR light‐base gene regulation strategies have been developed to modulate the fate of stem cells and improve the efficacy of the regenerative therapy.^[^ 27, 127, 128 ^]^

**Figure 11:**
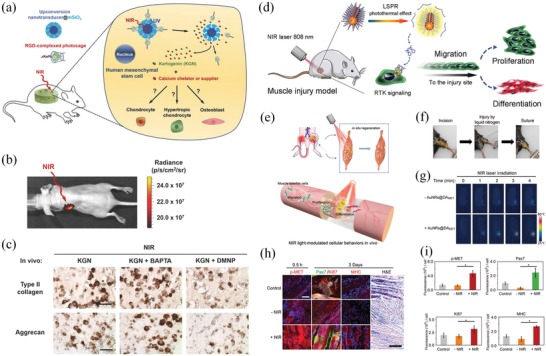
UCNP and Au‐based nanotransducers for NIR photoregulation of cell differentiation. a) Schematic illustration of NIR‐triggered chondrogenic differentiation of hMSCs after subcutaneous implantation in vivo. The release of KGN and/or either calcium chelator or calcium supplier caged in UCNT‐based nanocomplex was controlled by NIR illumination, thus regulating stem cell differentiation. b) In vivo tracking of subcutaneously implanted hMSCs in live mouse by NIR fluorescence imaging. c) Immunohistochemical assay of chondrocyte markers (Type II collagen and Aggrecan) at 21 d after hMSCs implantation. Scale bars represent 100 µm. Reproduced with permission.^[^ 127 ^]^ Copyright 2018, Wiley‐VCH. d) The NIR light‐activated DNA agonist (NIR‐DA) nanodevice for nongenetic manipulation of cell proliferation and differentiation via the receptor tyrosine kinase (RTK) signaling. e) Scheme of NIR‐DA system for modulating cellular behaviors of muscle satellite cells in the acute muscle injury animal model. f) The experimental procedure for preparing acute muscle injury animal model. g) The assay of photothermal effect of NIR light in mice. The 808 nm laser with 1.00 W cm^−2^ was used. h) Immunofluorescent analysis of p‐MET, Pax7, Ki67, and MHC protein in the sections from injured mice. Scale bar indicates 50 µm. i) Quantification of p‐MET, Pax7, Ki67, and MHC in the immunofluorescent images of (h). Reproduced with permission.^[^ 27 ^]^ Copyright 2019, American Chemical Society.

For example, Kang and colleagues developed an upconversion nanotransducer (UCNT)‐based nanocomplex for modulating stem cell differentiation in vitro and in vivo (**Figure** **11**a–c).^[^ 127 ^]^ In this study, 4‐(hydroxymethyl)‐3‐nitrobenzoic acid (ONA) and β‐cyclodextrin (β‐CD) were serially conjugated to the UCNT as a photolabile molecule and a caging molecule, respectively. Then, the chondro‐inductive kartogenin (KGN) and/or either calcium chelator or calcium supplier were caged in the UCNT. Upon NIR illumination, the UV light generated by the UCNT released the cargo molecules, thus real‐time regulating intracellular calcium to modulate stem cell differentiation (Figure 11a). By releasing different cargo molecules, hMSCs were successfully differentiated into chondrocytes, hypertrophic chondrocytes, or osteoblasts (Figure 11c). Moreover, the UCNT‐based nanocomplex could also be used to track the transplanted hMSCs in vivo (Figure 11b). In another interesting study, Li and colleagues developed a NaYF_4_:Yb^3+^Tm^3+^@SiO_2_ UCNP‐based strategy to induce and monitor stem‐cell differentiation.^[^ 128 ^]^ In their work, the photoactivatable caged siRNA and the MMP13 cleaved imaging peptide‐tetrapheny‐lethene (TPE) were conjugated within the mesoporous silica‐coated UCNPs. Upon NIR light stimulation, siRNAs were released from UCNPs to promote the osteogenic differentiation of hMSCs. After that, the TPE probe peptide was effectively cleaved by the MMP13 enzyme triggered by osteogenic differentiation, thereby real‐time reporting the differentiation of hMSCs by aggregation‐induced emission.

In addition to UCNT‐based photoregulation, localized photothermal stimulation mediated by photothermal nanomaterials can also be used to control cell behaviors by regulating intracellular gene transcription. In 2019, Wang and colleagues prepared a novel NIR light activated DNA agonist (NIR‐DA) nanodevice by coupling pre‐inactivated DNA agonists to AuNRs (Figure 11d–i).^[^ 27 ^]^ Under NIR illumination, the DNA agonist was released and activated through the photothermal effect of AuNRs. Then, the activated DNA agonist dimerized the DNA‐modified chimeric or native receptor tyrosine kinase (RTK) on the cell surface, thereby activating downstream signals in living cells. This NIR‐DA activation of the RTK signal controls cytoskeletal remodeling, cell polarization and directional migration, providing a powerful and versatile platform for exogenous regulation of cell differentiation in deep tissues. In the acute muscle injury animal model, the NIR‐DA system was successfully used to modulate the migration, proliferation, and differentiation of muscle satellite cells, thus helping the regeneration of injured muscle. In another interesting study, Ambrosone and colleagues developed a polyelectrolyte multilayer microcapsule to remotely manipulate the Wnt/β‐catenin signaling pathway in cells with NIR light.^[^ 129 ^]^ In this strategy, AuNPs in the polyelectrolyte multilayer microcapsule were used to generate heat after NIR illumination. Thereby, the encapsulated ALP was released to activate the Wnt pathway due to the heat‐induced disintegration of microcapsule. Considering the crucial role of the Wnt pathway in cell differentiation and fate, this strategy may be broadly translated to achieve remote manipulation of cell fate.

In addition to photothermally controlled drug release, photothermal stimulation also can directly regulate cell functions by modulating the hot‐related gene expression. In 2017, Marino and colleagues combined gold nanoshells (AuNSs) and NIR radiation to remotely stimulate striated muscle cells through photothermal conversion of AuNSs.^[^ 130 ^]^ It was found that the long‐term remote photothermal stimulation significantly increased mRNA transcription of genes encoding heat shock proteins and sirtuin 1. And the enhanced sirtuin 1 could further induce mitochondrial biogenesis in cells. The remote NIR excitation strategy has great potential in the fields of muscle tissue engineering and regenerating. In another example, Wang and colleagues synthesized a polymer nanoparticle with excellent photothermal conversion ability for controlling the target gene expression in cells.^[^ 49 ^]^ In their study, the heat‐inducible heat shock protein‐70 promoter was used to drive the expression of target genes. Upon the NIR illumination, the generated local heat activated the heat shock protein‐70 promoter and triggered the expression of targeted genes. This strategy used NIR light to control the expression of specific gene and has important applications in regenerative medicine.

#### 3.2.2 NIR Light‐Induced Gene Editing for Regenerative Medicine

Recently, the NIR light‐controlled CRISPR‐dCas9 has been successfully developed for gene transcription manipulation during tissue regeneration. For example, Shao and colleagues successfully engineered a light‐activated CRISPR‐dCas9 effector device to promote differentiation of induced pluripotent stem cells into neurons.^[^ 131 ^]^ This light‐activated CRISPR‐dCas9 effector device contained the dCas9 and the light‐sensitive bacterial phytochrome BphS. Upon light (≈730 nm) illumination, the activated BphS converted GTP into c‐di‐GMP, thus producing the FRL‐dependent transactivator p65‐VP64‐NLS‐BldD. Thereby, the transactivator bound to its chimeric promoter PFRLx to initiate expression of the light‐inducible genome transactivator (FGTA4). Further, the FGTA4 was recruited by the MS2 box of the sgRNA‐dCas9 complex to promote target gene expression. By using this novel device, the authors successfully up‐regulated the NEUROG2 (a neural transcription factor) in cells to promote differentiation of induced pluripotent stem cells into neurons. This strategy enables transcriptional activation of user‐defined genes under light illumination with precise spatiotemporal resolution, which will greatly boost the clinical progress of optogenetics‐based therapy in regenerative medicine.

#### 3.2.3 NIR Light‐Induced Photochemical Reactions for Regenerative Medicine

**Figure 12:**
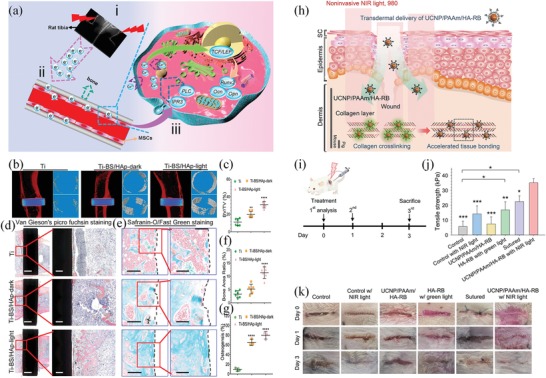
NIR light‐induced photochemical reactions for regenerative medicine. a) Scheme of NIR light‐activated Ti‐BS/Hap for osteogenic differentiation and bone regeneration. b) Micro‐CT 2D and 3D images of bone formation around the different implants. c) The quantitative assay of micro‐CT 3D images. d) Van Gieson's picro‐fuchsin staining of the bone sections. Blue: the nucleus of the osteoblast. Red: the bone. (scale bars = 1 and 100 µm). e) Safranin‐O/Fast Green staining of the bone sections. Green: the osteogenesis. Red or orange: the cartilage. (scale bars = 200 and 100 µm). f) Histomorphometric measurements of bone area rate. g) Histomorphometric measurements of osteogenesis. Reproduced with permission.^[^ 54 ^]^ Copyright 2019, American Chemical Society. h) Scheme of NIR light‐activated UCNP/PAAm/HA‐RB conjugate complex for photochemical tissue bonding of incised collagen matrix. i) The experimental setup for in vivo photochemical tissue bonding test in mice. j) In vivo tensile strength of the six groups at day 3. k) Images for the incised dorsal skin of BALB/c mice after different treatments. Reproduced with permission.^[^ 55 ^]^ Copyright 2017, American Chemical Society.

In recent years, photoregulation strategies using the mechanism of NIR light‐induced photochemical reactions have also been exploited for tissue engineering and regenerative medicine. In 2019, Wu and coworkers developed a photoelectric‐responsive extracellular matrix to control cell fate and improve bone regeneration (**Figure** **12**a–g).^[^ 54 ^]^ They produced a bismuth sulfide/hydroxyapatite (BS/HAp) film to create a fast and repeatable photoelectric‐responsive microenvironment around an implant. The photocurrent on the BS/HAp film was activated by NIR light (λ_ex_ = 808 nm), owing to the depletion of holes through PO_4_^3−^ from HAp and interfacial charge transfer by HAp compared with BS. Then, the photogenerated electrons transferred to the membrane of MSCs to change the concentration of sodium in intracellular and extracellular locations, resulting in a change in the membrane potential. Meanwhile, the Ca^2+^ inflow from the outside of the MSCs to the intracellular mitochondria via this potential difference and the increased Ca^2+^ further upregulated FDE1 and eventually arrived the cell nucleus to regulate the TCF/LEF. Furthermore, the TCF/LEF in the cell nucleus started transcription to regulate the downstream genes related with osteogenic differentiation, which was accomplished through the Wnt/Ca^2+^ signaling pathway. This study offered a novel strategy to noninvasively control cell differentiation behaviors by changing the photoelectric microenvironment in vivo using NIR light.

In another study, Han et al. developed a poly (allylamine) (PAAm) modified up‐converted nanoparticles/hyaluronic acid‐rose bengal (UCNP/PAAm/HA‐RB) conjugate complex for noninvasive partial‐thickness burn via the illumination of NIR light (λ_ex_ = 980 nm) (Figure 12h–k).^[^ 55 ^]^ The conjugate complex was transmitted deeply and widely through the skin from the boundary of incision due to the presence of HA in the outer layer. The UCNP of NaYF_4_:Yb/Er (Y:Yb:Er = 78:20:2) converted NIR to green light to activate RB encapsulated in HA‐RB conjugate, inducing the cross‐linking of collagen and promoting seamless tissue adhesion. This complex of UCNP/PAAm/HA‐RB conjugates might be broadly applied for the development of various phototherapy in regenerative medicine.

### 3.3 NIR Light for Neuromodulation

Neuromodulation is a crucial technique that can be applied in many fields such as the fundamental research of neural circuits, treatment of neurological diseases, and brain function manipulation.^[^ 43, 48, 141 ^]^ In recent years, light‐based neuromodulation has attracted great attentions due to its unique advantages of noninvasive and being capable of real‐time manipulating nerve cells in free‐moving animals with exceptional spatiotemporal precision. Currently, optogenetics is the most widely used technology for neuromodulation. Generally, the regulation of cellular physiological state under illumination can be achieved by exogenous expression of a genetically encoded photosensitive microbial ion channel protein, such as ChR,^[^ 44, 47 ^]^ Arch,^[^ 45 ^]^ and NpHR.^[^ 46 ^]^ In these optogenetic systems, ChR and Arch are used for neuronal activation, while Cl^−^‐transporting NpHR is used for inhibition of action potential production. However, these traditional optogenetic systems generally respond to visible light in the range of 430–630 nm, with a shallow tissue penetration depth. The development of red light sensitive ReaChR^[^ 47 ^]^ and NIR light‐sensitive BphPs^[^ 34 ^]^ proteins has made it possible to perform optogenetic research using red or NIR light with a wavelength of 620–720 nm. For example, Gomelsky and colleagues developed a Bph‐based system (IlaM5) for mammalian optogenetic applications. By using a transcranial irradiation of 730 nm, the brain activities of living mice were successfully controlled via the IlaM5‐based optogenetics. However, the tissue penetration of light with such a wavelength did not meet the needs of deep brain stimulation. More recently, the development of NIR light activatable nanomaterial‐mediated optogenetics have greatly extended the application of light for neuromodulation in deep tissues, such as the UCNP‐mediated optogenetics^[^ 48 ^]^ and photothermal nanomaterial‐mediated photothermal neuron activation.^[^ 52 ^]^

**Figure 13:**
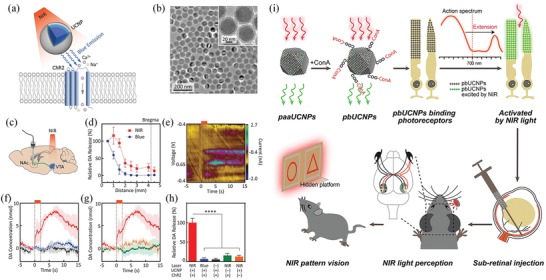
UCNP‐based optogenetics for neuromodulation. a) Schematic illustration of UCNP‐mediated NIR upconversion optogenetics for deep brain stimulation. b) TEM images of the blue‐emitting NaYF_4_:Yb/Tm@SiO_2_ UCNPs. c) Scheme of in vivo fast‐scan cyclic voltammetry (FSCV) to evaluate the efficacy of NIR‐evoked excitation of ventral tegmental area (VTA) in ventral striatum. d) Assay of DA release in ventral striatum under NIR and blue‐light stimulation. e) A trace of background‐subtracted current measured by FSCV in the ventral striatum of mouse after NIR stimulation (15 ms pulses at 20 Hz, 700 mW peak power). f,g) Transient DA concentrations in ventral striatum in response to transcranial VTA stimulation under different conditions. Each color corresponds to a stimulation condition shown in (h). h) Quantitative analysis of cumulative DA release within 15 s after stimulation under different conditions. Reproduced with permission.^[^ 28 ^]^ Copyright 2018, American Association for the Advancement of Science. i) The ocular injectable pbUCNPs for creating NIR light image vision in mice. pbUCNPs in PBS were injected into the subretinal space of the eyes and bound to the photoreceptors in the mouse retina. The retinal pbUCNPs can transform mammalian invisible NIR light into visible emissions with short wavelength, thus helping the mice to acquire NIR light image vision. Reproduced with permission.^[^ 135 ^]^ Copyright 2019, Elsevier Inc.

The most commonly used NIR light‐responsive nanomaterial is UCNPs that can convert NIR light into UV or visible light, which in turn excites traditional opsin proteins to activate or inhibit cellular signals. For example, Han et al. synthesized dye‐sensitized core/active shell UCNPs to activate ReaChR‐expressed hippocampal neurons with 800 nm NIR light.^[^ 133 ^]^ In this system, ytterbium ions (Yb^3+^) was doped in the UCNP shell to bridge the energy transfer from the IR806 dye to the UCNP core, which bridged the energy transfer from the dye to the UCNP core. In this way, the energy of NIR excitation light (800 nm) was absorbed by IR‐806 dyes and was effectively transferred to the UCNP core, thus significantly enhancing the upconversion luminescence of UCNPs. By using the dye‐sensitized UCNPs, the activity of ReaChR‐expressed hippocampal neurons was controlled by 800 nm NIR light with deep tissue penetration, minimal heating and high spatiotemporal precision. In another interesting study, Ao and colleagues studied the movement behavior control of *Caenorhabditis elegan* by Yb^3+^/Er^3+^/Ca^2+^‐based lanthanide‐doped UCNPs.^[^ 134 ^]^ They found that the uranium‐doped UCNP effectively activated Chrimson (a green‐activated cation channel) and inhibited GABAergic motor neurons during 808 nm NIR light irradiation, thereby reducing the action potential of body wall muscle discharge and inhibiting the movement of nematodes. Thanks to the deep penetration of NIR‐Ⅱ light (1000–1700 nm), UCNP‐mediated optogenetics have also been used to spur deep brain neurons in living animals. In 2018, Chen et al. synthesized blue‐emitting NaYF_4_:Yb/Tm@SiO_2_ UCNPs for optical manipulation of neuronal activity in the deep brain (**Figure** **13**a–h).^[^ 28 ^]^ In this study, the authors injected these UCNPs into the ventral tegmental area of the mouse brain. After NIR light (980 nm) irradiation at a distance of a few millimeters outside the skull, the channel rhodopsin expressed in dopaminergic neurons was activated and a rapid increase in dopamine release in neurons was observed (Figure 13c–h). In another case, UCNPs were also used to inhibit hippocampal (HIP) activity during chemically induced seizure via simple transcranial NIR irradiation. These studies suggested that UCNP‐mediated optogenetics is a flexible, powerful and minimally invasive nanotechnology‐assisted method for optically controlling neuronal activity in vitro and in vivo.

In addition to modulating the activity of neurons, UCNPs were also used to help mammalian to acquire NIR light image vision. It is well known that mammals cannot see light with a wavelength exceeding 700 nm. In 2019, the Xue and coworkers developed ocular injectable photoreceptor‐binding upconversion nanoparticles (pbUCNPs) to break this barrier (Figure 13i).^[^ 135 ^]^ The synthesized pbUCNPs acted as a miniature NIR light sensor anchored to the photoreceptor of the retina, allowing the mouse to produce a more accurate NIR light image. More interestingly, animals injected with pbUCNP not only sensed NIR and visible light patterns at the same time, but also accurately distinguished NIR images. This new approach will undoubtedly provide new ideas for the design and application of emerging bio‐integrated nanodevices.

In addition to UCNPs, photothermal nanomaterials have also been developed for neuronal activity manipulation, including Au, semiconducting polymer and Si nanomaterials.^[^ 52, 142, 143 ^]^ Because most native nerve cells are sensitive to the local temperature, these photothermal neuromodulation methods generally do not require genetic modification of target cells. In 2014, Yong and colleagues investigated the application of silica‐coated AuNRs as an extrinsic absorber of NIR light in cells to stimulate the activity of neurons.^[^ 52 ^]^ They found the electrical activity of the NR‐treated auditory neurons significantly increased after stimulation by a NIR laser at 780 nm. The NIR laser stimulation induced temperature changes of between 0.5 and 6.0°C in the neurons correlated well with the electrical activity of the neurons, suggesting the photothermal effects of AuNRs contributed to neuromodulation by the NIR laser. In another example, Carvalho‐de‐Souza and colleagues found that cell‐membrane targeted AuNPs could transduce millisecond pulses of light into heat, subsequently inducing membrane capacitance changes and depolarizing the cell.^[^ 143 ^]^ These studies suggested that different cell phenotypes can be targeted by AuNPs with specific ligands and specifically activated by NIR light, thus serving as an alternative strategy to selective optical stimulation of specific cells in tissue. However, the application of photothermal materials may induce potential cellular thermal damage, which is still not fully understood and resolved.

In addition to AuNPs, biodegradable organic polymer nanomaterials have also been used in neuromodulation research due to their good biocompatibility and excellent optical properties.^[^ 117, 136 ^]^ In a recent work, Pu et al. developed semiconducting polymer nanobioconjugates (SPNsbc) for controlling the transient receptor potential cation channel subfamily V member 1 (TRPV1) in neurons.^[^ 136 ^]^ The synthesized SPNsbc possessed an efficient NIR (808 nm) absorbing capability and a high photothermal conversion efficiency, which served as a photothermal nanomodulator to control the thermosensitive ion channels in neurons. Moreover, the TRPV1 antibody on the surface of SPNsbc enabled SPNsbc to precisely target the TRPV1 ion channel on the surface of neuronal membranes, thereby achieving precise regulation of TRPV1 ion channels with minimal off‐target side effect and probability of noxious heat to living cells. In addition, the fast heating capability and good photothermal stability of SPNsbc enabled SPNsbc to control the intracellular Ca^2+^ influx of neurons by NIR irradiation at 808 nm within milliseconds in a reversible manner.

Another interesting nanomaterial for nongenetic neuromodulation is the light‐sensitive silicon nanostructure developed by Tian et al.^[^ 141, 144 ^]^ In their studies, silicon nanostructure can excite neural activities via a rapid photothermal process^[^ 142 ^]^ or photoelectrochemical reaction^[^ 141, 144 ^]^ during a pulse light illumination. In addition, they have developed a set of silicon materials (such as Si nanowire, Si membrane, and Si mesh) for different biomedical applications, including intracellular calcium dynamics modulation, cellular excitability manipulation, cytoskeletal structures and transport regulation and brain activity control. Although these studies were mostly performed using the 532 nm laser pulses, this strategy might be further extended to NIR light since silicon is also capable of absorbing light in the infrared regime.

The development of NIR light‐based neuromodulation techniques has offered a possibility of real‐time controlling neuronal activity at the single cell or subcellular level with the flexibility of optical stimulation, which will have great potential for fundamental neuroscience research and neurological disease treatment.

## 4 NIR Light for Simultaneous Cell Sensing and Modulation

The simultaneous monitor and modulation of cell functions in vivo have wide applications in precision medicine. Taking the diseases treatment as an example, light‐based disease treatment, and therapeutic efficacy evaluation could be simultaneously achieved in real time, allowing precisely guide the disease treatment process. On the other hand, the target‐responsive imaging can accurately report lesions and guide the precise light therapy of diseases. Recently, the dual‐functional strategies have been successfully applied in cancer diagnosis and therapy.^[^ 66, 67 ^]^

For example, apoptosis imaging techniques were combined with cancer therapy strategies to achieve cancer treatment and simultaneous therapeutic efficacy evaluation. Min et al. developed a cancer therapy and diagnosis system by conjugating a fluorophore–DEVD–quencher pair (Cy5‐DEVD‐Qsy21) to silica‐coated UCNPs. Thus, the fluorescence of Cy5 activated by caspase was used to real‐time report the therapeutic efficacy of NIR light‐activatable UCNP‐platinum(IV) prodrug.^[^ 59 ^]^ In a similar manner, Zhang and colleagues proposed a NIR‐II PTT and real‐time apoptosis imaging strategy by loading peptide DTPP (PpIX‐PEG8‐GGKGRDEVDGC) on the surface of Au hollow nanorods (AuHNRs). The quenched fluorescence of Potosensitizer (PpIX) in AuHNRs was turned on when exposed to caspase‐3, reporting the therapeutic efficacy of PDT for tumor.^[^ 50 ^]^

**Figure 14:**
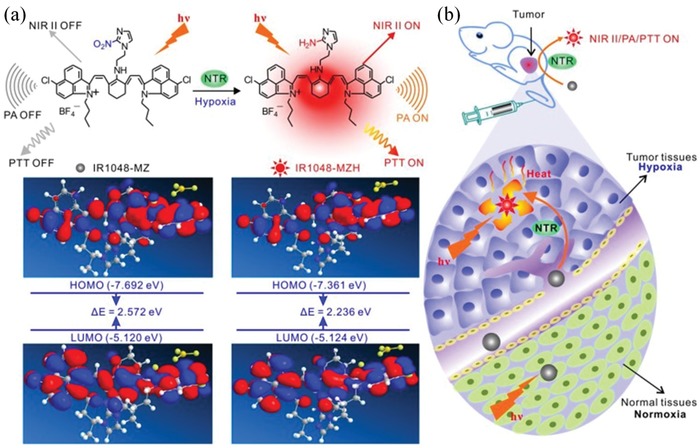
The hypoxia‐triggered NIR‐II fluorescence probe for tumor diagnosis and PTT. a) The mechanism of IR1048‐MZ activated by nitroreductase in cells. b) The NIR‐II/PA/PTT of IR1048‐MZ was quenched in the normal tissues while activated by NTR in cancer cells and hypoxic tumors. Reproduced with permission.^[^ 67 ^]^ Copyright 2018.

For imaging‐guided cancer therapy, Tian and colleagues developed a hydrogen sulfide (H_2_S)‐activatable probe (Nano‐PT) for NIR‐II fluorescence‐guided PTT of colorectal cancer.^[^ 66 ^]^ In their study, the synthesized Nano‐PT contained a monochlorinated BODIPY core that could be served as an H_2_S‐activatable unit through the thiol‐halogen nucleophilic substitution between BODIPY and H_2_S. In addition, the Nano‐PT was also served as a photothermal agent with a high light‐to‐heat energy conversion capability. With the HCT116 tumor‐bearing mice as a model, the NIR‐II fluorescence of Nano‐PT was turned on in the tumor site and therefore guided the precise PTT of colorectal cancer. Hypoxia is a typical feature of solid tumors and an important factor of the radio‐chemotherapy resistance of tumors.^[^ 145, 146 ^]^ In 2018, Cai et al. developed a hypoxia‐triggered NIR‐II fluorescence probe for tumor imaging and PTT (**Figure** **14**).^[^ 67 ^]^ The synthesized probe (named IR1048‐MZ) contained a nitro imidazole group as a specific hypoxia trigger and an IR‐1048 dye as a signal reporter. Thereby, the NIR‐II fluorescence and photoacoustic (PA) signals of IR1048‐MZ was triggered in the tumor site, thus clearly reporting the boundary of tumors in deep tissues with high spatial resolution. Moreover, the probe also showed an enhanced photothermal effect for tumor therapy after being activated by the overexpressed nitroreductase in hypoxic tumors. Hence, the novel hypoxia‐triggered probe offered a good choice for simultaneous diagnosis and imaging‐guided precise PTT of tumors.

## 5 Conclusion and Perspectives

In this review, we provided a summary of recent progress with respect to the NIR light for monitoring and modulating the spatiotemporal dynamics of cell functions in living system. Generally, the low energy NIR light has lower phototoxicity and higher tissue penetration depth in living systems as compared with UV/vis light, and thereby is promising for in vivo applications.^[^ 8, 14, 15 ^]^ Moreover, due to the reduced photon absorption and scattering, minimal tissue autofluorescence of NIR light in tissues, the NIR light‐based imaging and photoregulation strategies have offered a possibility to real‐time sense and/or modulate specific molecular events in deep tissues with subcellular accuracy.^[^ 5, 40, 42 ^]^ Thereby, the NIR light‐based bioimaging and photoregulation not only improve our understanding on the underlying pathological mechanisms, but also aid to optimize therapeutic interventions in biomedical fields including cancer diagnosis and therapy, regenerative medicine, and neuroscience. Although important progress in bioimaging and photoregulation have been achieved, NIR light‐based bioimaging and photoregulation still face many challenges both in fundamental biomedical research and future clinical translation.

For NIR imaging, efforts to further improve the resolution, sensitivity, specificity of NIR imaging are urgently needed. First, there is rare of NIR‐II fluorophores. It is known that the NIR‐II imaging overwhelms the conventional fluorescence imaging using visible and NIR‐I fluorophores in terms of tissue penetration and spatiotemporal resolution.^[^ 8 ^]^ Therefore, the development of NIR‐II fluorophores with high brightness and high compatibility is urgent. Second, NIR probes are generally designed to monitor a specific cellular event, but accompanying with false signal interference from the unspecific cells. Thereby, the development of multiphysiological parameter‐activatable probes may be an effective strategy to reduce the nonspecific signal interference. Third, the occurrence and development of diseases are generally accompanied by changes in multiple interrelated physiological characteristics. Multichannel optical imaging is able to simultaneously monitor multiple cellular events in cells and tissues. Thus, the further development of multichannel optical imaging in the NIR window may be helpful for revealing the underlying mechanism of the complex disease development.

For in vivo photoregulation, NIR‐II light has greatly promoted the application of light‐based cell manipulation technologies in deep tissues. The emergence of a series of NIR light nanotransducers (such as UCNPs and AuNPs) that can convert NIR‐II light into visible/UV light or heat has made NIR‐II optogenetics possible.^[^ 5, 42 ^]^ However, several challenges are remained in fundamental biomedical research and clinical translation. First, the quantum yield of UCNPs and the photothermal conversion efficacy of Au nanomaterials are still relatively low. Thus, it is highly important to improve the photo conversion efficacy of NIR light transducers to reduce the power of NIR laser used in photoregulation, thereby improving the photoregulation efficacy to target cells and avoiding the possible heating effect of the NIR light to the non‐target cells. Second, the multichannel optogenetics that is capable of modulating multiple cellular events by lights with different wavelength has important applications in biomedical research. Nowadays, activation or inhibition of cell activity can be achieved by stimulating Arch or NpHR channel proteins with different visible light.^[^ 147, 148 ^]^ However, multichannel optogenetics using multiple NIR lights has not been achieved yet. Thereby, the development of NIR light‐based multichannel optogenetics will further promote the application of optogenetics in biomedicine. Third, most of the current optogenetics need to genetically engineer cells, which is not suitable for the future clinical application of optogenetics technology. In addition, the photothermal nanomaterial‐based nongenetic photoregulation techniques usually suffer from the limitations, such as the side effect of overheating, relatively slow response and unclear working mechanism.^[^ 52 ^]^ Most of the photoelectrochemical reaction‐based strategies are limited to the use of visible light.^[^ 141, 144 ^]^ The development of nongenetic photoregulation technique with high tissue penetration depth, high sensitivity and high safety is urgently needed for future clinical translation. Fourth, the potential toxicity of nanotransducers, such as the widely used AuNPs and UCNPs, should be taken into concern. To develop biocompatible and biodegradable nanotransducers is greatly appreciated for the future clinical applications of phototherapy.

In addition to the above challenges, the combination of molecular imaging and photoregulation techniques for simultaneous monitor and modulation of cell functions in vivo will have great applications in precision medicine but also remain challenge. At present, the dual‐functional strategy has only been tested in few cases, such as cancer diagnosis and therapy.^[^ 66, 67 ^]^ Thereby, more strategies for simultaneous cell sensing and modulation should be designed and developed to meet the needs in different research fields, including regenerative medicine and neuroscience. In addition, further efforts are also needed to improve the sensitivity, specificity, and safety of the multifunctional light strategy to meet the needs of future clinical applications.

For both cell function sensing and modulation, the wavelength of the light is a critical factor involved in the efficiency of sensing and modulation. Therefore, the role of wavelength in biomedicine applications of light also needs to be further explored. At present, NIR light with a wavelength of 700–1700 nm is the most widely used light in biomedicine partially due to the lowest absorption by water and the deepest tissue penetration depth of light in this rang compared with light in other IR range. Recently, NIR laser with a wavelength of 2100 nm has been used in nerve stimulation researches.^[^ 149, 150 ^]^ Meanwhile, mid‐infrared radiation (MIR, 3.0–50 µm) and far‐infrared radiation (FIR, 50–1000 µm) also have great potential for biomedical applications, such as wound healing, heart failure therapy, and laser surgery.^[^ 151, 152 ^]^ As compared with NIR light, light in the MIR and FIR range are more easily absorbed by water and other components in the tissue, thus possessing a shallow tissue penetration depth. This is the main factor that limits the application of MIR and FIR in deep tissue imaging and regulation. In addition, the exact mechanisms of effects of MIR and FIR irradiation on biological activities are still poorly understood, which further limit their biomedical applications. Therefore, the further understanding the mechanisms (both the thermal effects and non‐thermal effects) involved in the IR‐tissue interactions may help to the development of MIR and FIR light‐based strategies for biomedical applications. Moreover, the development of light conversion materials and technologies that can transform MIR or FIR into detectable signals (such as ultrasound signal) with high tissue penetration depth or high cellular response will greatly expand the biomedical applications of MIR and FIR light.

Overall, the NIR light‐based strategies have offered a great possibility of monitoring and modulating cells with a subcellular and sub‐millisecond accuracy in deep tissues, which are inaccessible to other technologies. We expect that the research along this direction may lead to the development of innovative technologies for clinical theranostics in the near future.

## Conflict of Interest

The authors declare no conflict of interest.
